# Supplementary material for: Effect of the Silica–Magnetite Nanocomposite Coating Functionalization on the Doxorubicin Sorption/Desorption
Source: Pharmaceutics. 2022 Oct 24;14(11):2271. doi: 10.3390/pharmaceutics14112271 (PMC9694706; doi:10.3390/pharmaceutics14112271)
Supplement: Supplementary file 1 [file pharmaceutics-14-02271-s001.zip › pharmaceutics-1954130-supplementary.pdf]

Supplementary Materials

Effect of the Silica–Magnetite Nanocomposite Coating Functionalization on the Doxorubicin Sorption / Desorption

Alexander M. Demin [1,*], Alexander V. Vakhrushev [1], Marina S. Valova [1], Marina A. Korolyova [1], Mikhail A. Uimin [2], Artem S. Minin [2], Varvara A. Pozdina [3,4], Iliya V. Byzov [2], Andrey A. Tumashov [1], Konstantin A. Chistyakov [1], Galina L. Levit [1], Victor P. Krasnov [1,*] and Valery N. Charushin [1,5]

[1] Postovsky Institute of Organic Synthesis, Russian Academy of Sciences (Ural Branch), Ekaterinburg 620108, Russia
[2] Mikheev Institute of Metal Physics, Russian Academy of Sciences (Ural Branch), Ekaterinburg 620990, Russia
[3] Institute of Immunology and Physiology, Russian Academy of Sciences (Ural Branch), Ekaterinburg 620049, Russia
[4] Institute of Natural Sciences and Mathematics, Ural Federal University, Ekaterinburg, 620002, Russia
[5] Institute of Chemical Engineering, Ural Federal University, Ekaterinburg 620002, Russia

Estimation of Inorganic Component Ratio (SiO_2 : Fe_3O_4) Based on the UATR-FTIR Data

The estimations of inorganic component ratio (SiO_2 : Fe_3O_4) were performed based on the UATR-FTIR data. For this, we used Omnic 7.3 software. Figure S1 shows a scheme for calculating the areas of absorption bands related to vibrations of Si–O and Fe–O bonds in the UATR-FTIR spectra of NC **6** as an example. The original reflectance spectrum (Figures 2 and S1a) was transformed into an absorption spectrum (Figure S1b). Next, to calculate the area ratio of the absorption bands, we chose the bands of vibrations of Si–O bonds in the range of 1300–755 cm^{-1} (Figure S1c) and the bands of vibrations of Fe–O bonds in the range of 750–500 cm^{-1} (Figure S1d).

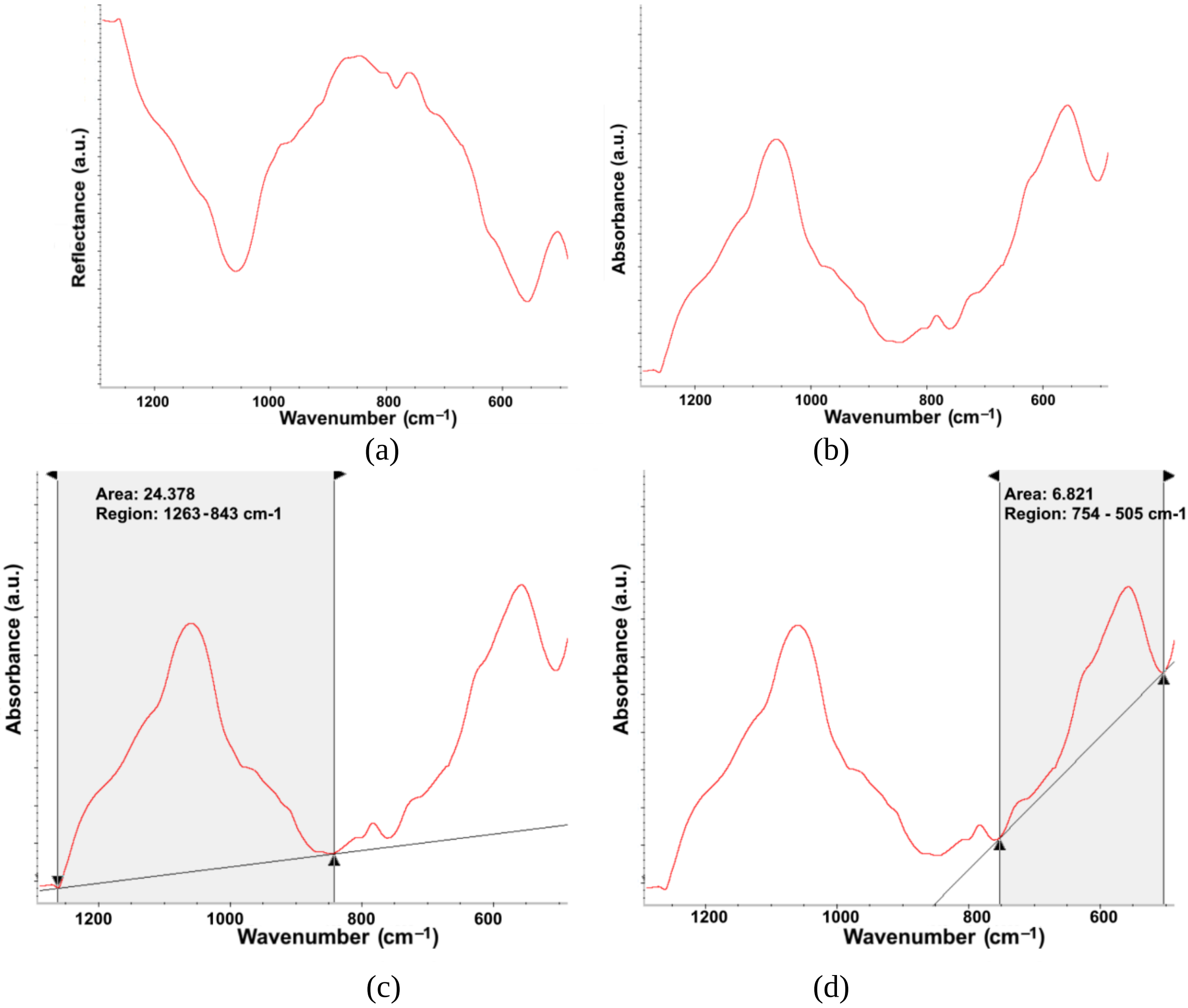

Figure S1. Calculation of areas of absorption bands related to vibrations of Si–O and Fe–O bonds in UATR-FTIR spectra of NC **6**: (a) reflectance and (b) absorbance spectra; calculation of areas of absorption bands related to vibrations of (c) Si –O and (d) Fe–O bonds.

Magnetization Reversal Curves

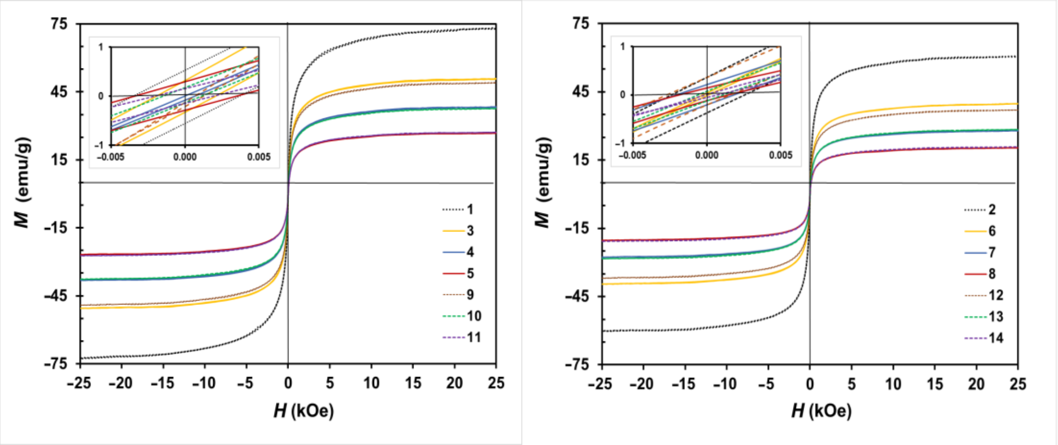

Figure S2. Magnetization reversal curves for NCs **1, 3–5, 9–11** (left) and **2, 6–8, 12–14** (right).

TEM Analysis

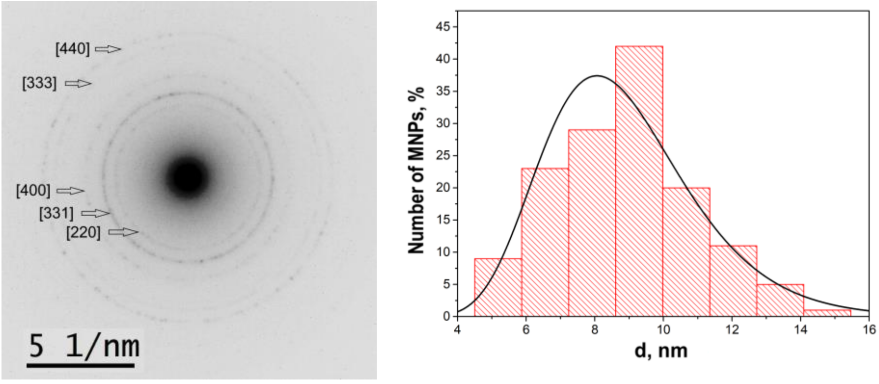

(a) (b)

Figure S3. (a) Electron diffraction pattern of NC **18** (corresponds to JCPDS Card No. (79 - 0417) Magnetite – synthetic). (b) The size distribution of Fe_3O_4 MNPs (core) in composition of NC **18** from TEM data.

Core size histogram was constructed using the Sturges' method (Figure S3b). To do this, we used the data for 143 nanoparticles (N = 143). The bin-width (W) is obtained from the formulas (S1) and (S2):

$W = (D_{max} - D_{min})/k$, (S1)

$k = 1 + 3.322 \times \log(N)$, (S2)

where D_{max} = 15.45 nm, D_{min} =4.45 nm, k is the number of bins in histogram, N is the number of nanoparticles used in calculations.

A core size histogram obtained in Origin 2022 software can be considered as a log-normal distribution. A mean particle size (D_{mean}) and the dispersity (σ) were 8.8 and 0.24 nm, respectively.

UV-Vis and Fluorescence Spectrometry

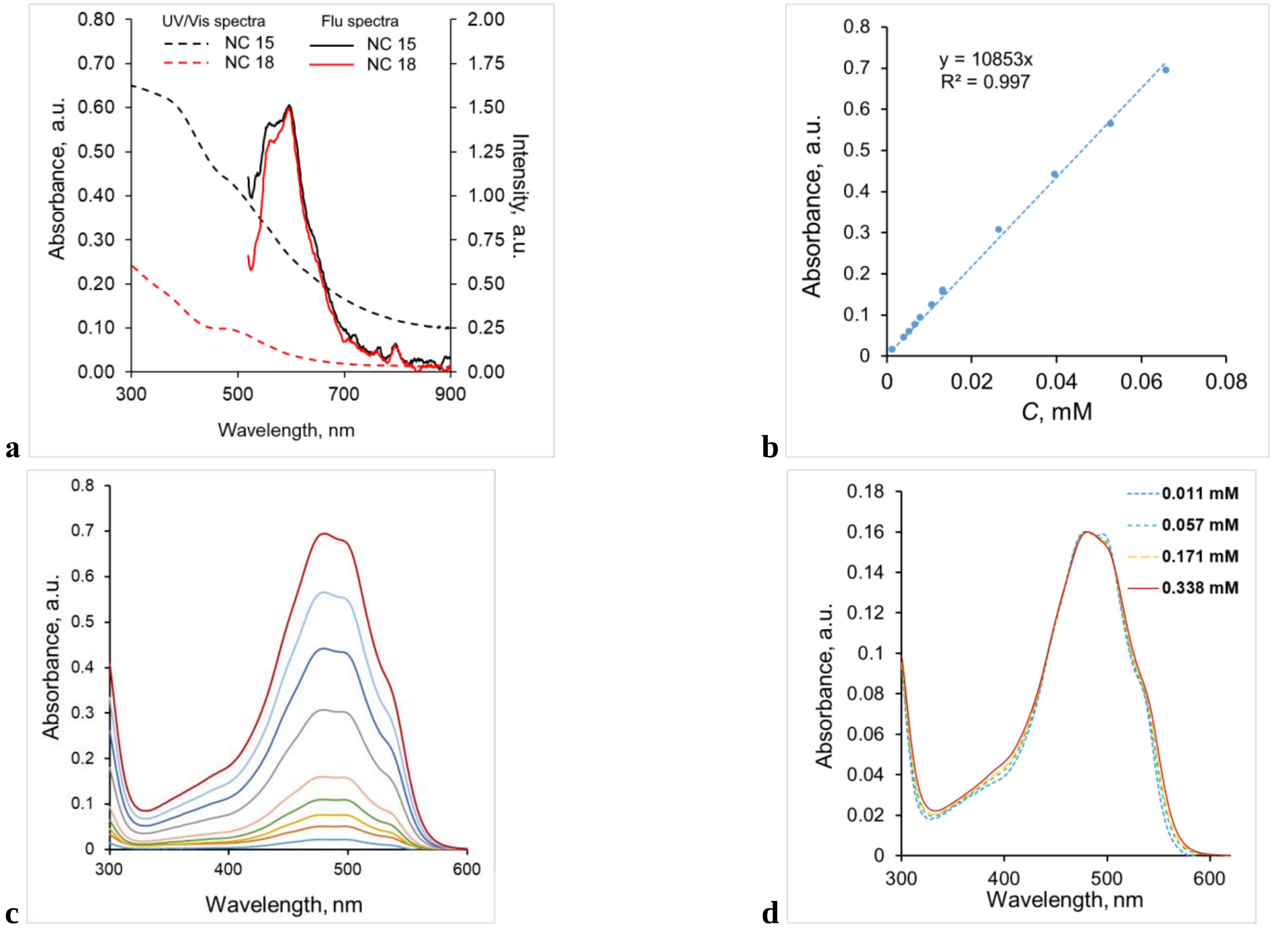

Figure S4. (**a**) Absorption and fluorescence spectra of colloidal solutions of NCs **15** and **18**. (**b**) Determination of the molecular extinction coefficient of Dox at 480 nm, concentration from 2 × 10^{-6} to 7 × 10^{-5} M. (**c**) Fragment of the absorption spectra of analyzed Dox solutions in the range of 300–600 nm. (**d**) Normalized absorption spectra of Dox at various concentrations.

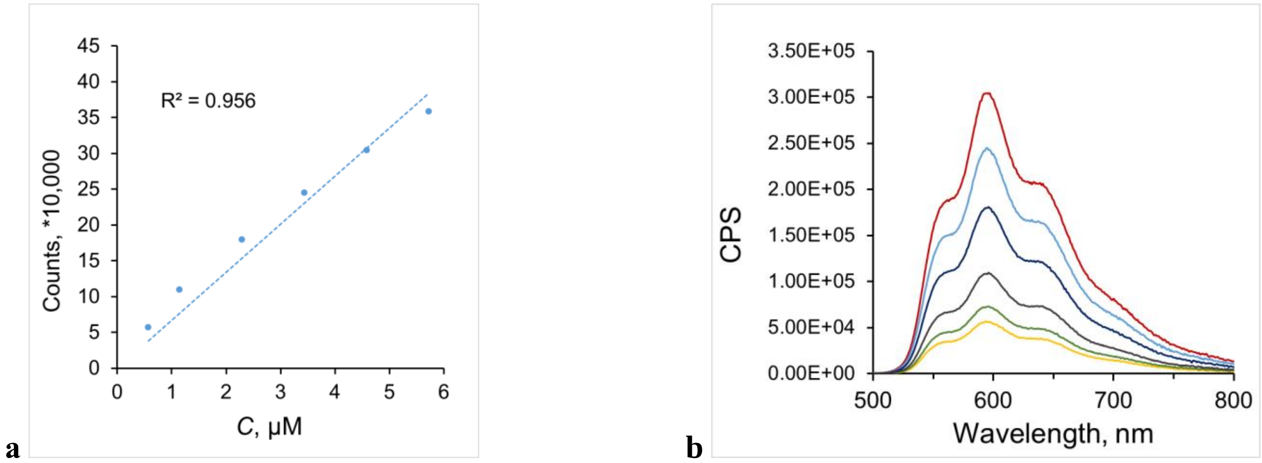

Figure S5. (**a**) Calibration plot of fluorescence intensity at 594 nm *vs*. Dox concentration. (**b**) Fragment of the emission spectra of analyzed Dox solutions in the range of 500–800 nm.

Dox Sorption onto MNPs 1 and 2

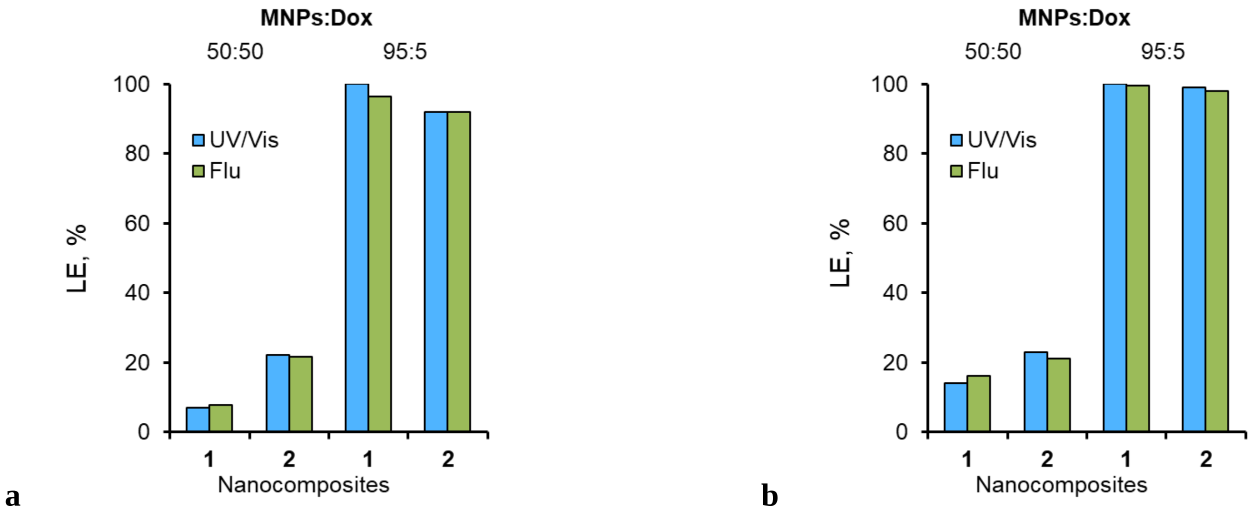

Figure S6. The efficiency of Dox sorption onto MNPs **1** and **2** for (**a**) 1 min and (**b**) 24 h; MNPs–Dox ratio 50:50 or 95:5. Dox loading efficiency (LE, %) was calculated from the UV-Vis and fluorescence spectrometry data.

Sorption proceeds rather quickly; the LE values of the samples isolated 1 min and 24 h after the beginning of sorption differed insignificantly. At a MNPs–Dox ratio of 95:5, LE for both types of particles reached 100%. The calculated LE values for MNPs **1** and **2** were 7 and 23%, respectively.

Cartesian Coordinates of Dox, SiO_2 Cluster, Conjugates of SiO_2 Cluster with APS and PMIDA, and Their Dox Complexes with the Lowest Energy at the B3LYP-D3-gCP/def2-SVP Level of Theory.

E is total electronic energy in Hartree's

Dox

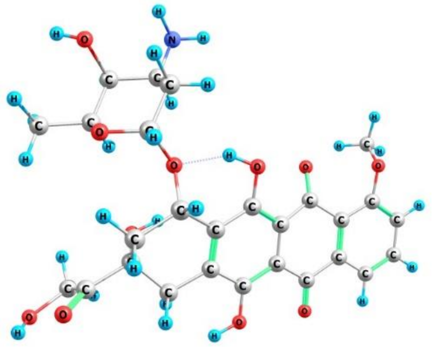

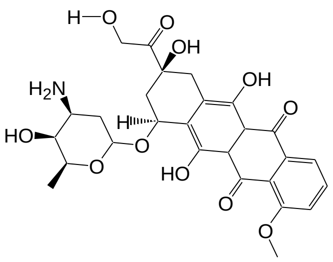

E = -1925.926799567

O	2.083051000	0.650286000	-0.763313000
O	4.336244000	1.197017000	-1.209375000
O	2.924900000	-1.431342000	1.573533000
O	5.171303000	4.051447000	-0.785732000
O	-0.518366000	0.947481000	-1.027788000
O	-1.039242000	-4.124797000	1.096376000
O	4.587831000	-3.932369000	-0.220220000
O	5.718316000	-4.394287000	2.157725000
O	-2.793151000	1.504020000	0.226843000
O	-3.613706000	-3.660533000	1.310114000
O	-5.437649000	1.840335000	-0.383846000
N	2.565119000	4.966279000	-0.243705000
C	1.781299000	-0.726311000	-1.116439000
C	2.894259000	-2.367294000	0.497604000
C	2.961217000	-1.687682000	-0.881115000
C	1.601743000	-3.227795000	0.524384000
C	0.488549000	-1.136036000	-0.402751000
C	0.373641000	-2.381581000	0.227612000
C	3.001967000	1.363266000	-1.622571000
C	2.605297000	2.845658000	-1.641105000
C	2.879444000	3.533956000	-0.282205000
C	4.351671000	3.296010000	0.111376000
C	4.679083000	1.782337000	0.073358000
C	-0.641841000	-0.270402000	-0.462311000
C	4.108295000	-3.296241000	0.702093000
C	-0.909069000	-2.863337000	0.622240000
C	-1.888630000	-0.690067000	0.044941000
C	-2.049042000	-2.030172000	0.518279000
C	6.153524000	1.474937000	0.308627000
C	4.696165000	-3.427443000	2.102928000
C	-3.008881000	0.299411000	0.173079000
C	-3.408933000	-2.527781000	0.884174000
C	-4.394903000	-0.247297000	0.303208000
C	-4.576597000	-1.601021000	0.673326000
C	-5.541704000	0.564788000	0.095751000
C	-5.867370000	-2.117480000	0.872457000
C	-6.825045000	0.029621000	0.298054000
C	-6.987588000	-1.302448000	0.691031000
C	-5.539209000	2.909740000	0.566299000
H	1.548473000	-0.743067000	-2.203797000
H	2.936120000	-2.475032000	-1.654050000
H	3.907430000	-1.138566000	-1.007849000
H	1.500344000	-3.690977000	1.525644000
H	1.706672000	-4.055572000	-0.204967000
H	2.944185000	0.927158000	-2.639512000
H	1.544647000	2.957072000	-1.937981000
H	3.207151000	3.337244000	-2.427944000
H	2.261360000	3.035268000	0.490286000
H	4.501282000	3.649079000	1.153877000
H	4.071409000	1.287221000	0.858426000
H	2.101203000	-0.913466000	1.587339000
H	6.332946000	0.385252000	0.279750000
H	6.779723000	1.949030000	-0.468420000
H	6.474902000	1.852723000	1.296850000
H	1.601949000	5.114297000	-0.564146000
H	3.157492000	5.450495000	-0.926813000
H	3.869499000	-3.670030000	2.807013000
H	5.061938000	-2.421983000	2.408617000
H	6.019287000	4.236547000	-0.350830000
H	0.440428000	1.178343000	-1.011562000
H	-0.187771000	-4.593988000	1.057776000
H	-5.979344000	-3.162550000	1.176644000
H	5.847516000	-4.717191000	1.243584000
H	-7.694966000	0.673080000	0.119762000
H	-7.994203000	-1.708918000	0.847501000
H	-6.511698000	2.889326000	1.098234000
H	-5.460989000	3.849005000	-0.007289000
H	-4.716719000	2.864674000	1.303581000

PMIDA

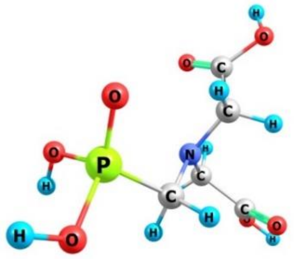

E = -1118.158188107

P	1.203739000	-4.492116000	10.641313000
C	2.699853000	-3.639592000	11.274591000
H	3.519238000	-4.390413000	11.316458000
H	2.998494000	-2.902083000	10.507353000
N	2.436144000	-2.959828000	12.538658000
C	2.396917000	-3.817456000	13.713843000
C	1.537299000	-3.241106000	14.838027000
O	1.828764000	-3.829208000	16.011528000
H	1.227895000	-3.479863000	16.701953000
O	0.687524000	-2.385123000	14.714880000
H	1.923157000	-4.780265000	13.440843000
H	3.399282000	-4.070795000	14.124085000
C	3.121959000	-1.693870000	12.713959000
C	4.650336000	-1.780137000	12.813999000
O	5.194421000	-0.568097000	13.010429000
H	6.168857000	-0.656665000	13.065167000
O	5.300476000	-2.800684000	12.720282000
H	2.749110000	-1.178461000	13.618873000
H	2.881439000	-1.009589000	11.876550000
O	0.591447000	-5.525158000	11.538813000
O	0.091147000	-3.355070000	10.286954000
H	0.391605000	-2.665112000	9.664821000
O	1.776607000	-5.050400000	9.222418000
H	1.151968000	-5.644950000	8.761833000

PMIDA-Dox

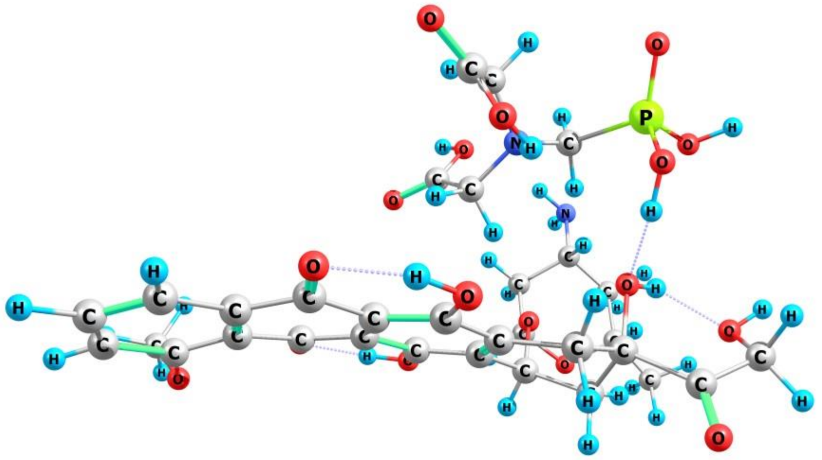

***E*=-3044.139790198**

P	1.161815000	-4.524886000	11.055868000
C	2.741884000	-3.661346000	11.430340000
H	3.555470000	-4.415911000	11.446184000
H	2.953252000	-2.983788000	10.584161000
N	2.636536000	-2.874547000	12.662380000
C	2.647835000	-3.622319000	13.921403000
C	1.522792000	-3.178310000	14.873060000
O	0.554305000	-2.452982000	14.294946000
H	0.807092000	-2.354919000	13.343229000
O	1.480573000	-3.480321000	16.045197000
H	2.465366000	-4.694478000	13.720739000
H	3.608709000	-3.557349000	14.471745000
C	3.369790000	-1.612788000	12.688992000
C	4.901068000	-1.651461000	12.813542000
O	5.457176000	-2.723798000	12.224421000
H	6.431604000	-2.679124000	12.324352000
O	5.540005000	-0.769859000	13.345615000
H	3.008973000	-0.987331000	13.524001000
H	3.147870000	-1.051814000	11.761234000
O	0.745427000	-5.604160000	12.011135000
O	0.022319000	-3.373833000	10.977851000
H	0.282876000	-2.583602000	10.421137000
O	1.479607000	-4.989318000	9.524496000
H	0.844178000	-5.644460000	9.174006000
O	3.408205000	0.154745000	9.083791000
O	4.080247000	0.688604000	6.879279000
O	5.773749000	-1.371510000	5.458317000
O	4.450074000	2.178326000	10.991064000
H	5.087470000	2.473187000	11.710137000
O	-0.341393000	0.813951000	13.323533000
H	-0.291816000	0.984851000	14.308340000
O	5.391196000	2.692526000	13.285590000
O	0.623569000	1.494336000	15.588836000
O	6.429663000	3.076034000	15.665885000
N	5.774391000	-3.174309000	7.652897000
C	2.459782000	1.212900000	9.309931000
H	2.888340000	2.165472000	8.940498000
C	0.238818000	-0.074644000	9.233891000
C	-1.012247000	-0.310439000	8.342363000
C	-0.993853000	-1.478675000	7.347308000
H	-1.670627000	-1.214584000	6.511507000
H	-1.445219000	-2.344487000	7.882991000
O	0.326618000	-1.771365000	6.893882000
H	0.319946000	-2.609467000	6.398795000
O	-2.001824000	0.387238000	8.434141000
O	0.917128000	-1.327181000	9.452176000
H	1.155624000	-1.686648000	8.567810000
C	1.150297000	0.968153000	8.542419000
H	0.583255000	1.916077000	8.484895000
C	-0.154850000	0.459015000	10.612460000
C	2.234979000	1.347780000	10.811422000
C	1.025108000	0.987156000	11.401559000
C	4.495789000	0.445917000	8.216472000
C	5.476691000	-0.729582000	8.305486000
C	4.909778000	-1.991221000	7.612733000
C	4.553201000	-1.639031000	6.155129000
C	3.576342000	-0.440648000	6.130412000
C	3.316075000	1.836785000	11.616319000
C	0.854235000	1.137814000	12.811835000
C	3.170785000	1.943734000	13.017825000
C	1.918713000	1.602599000	13.613275000
C	3.257293000	0.067438000	4.725494000
C	4.318443000	2.386358000	13.840213000
C	1.724123000	1.742352000	15.056235000
C	4.146925000	2.464163000	15.315344000
C	2.875910000	2.181192000	15.892480000
C	5.215669000	2.839774000	16.192652000
C	2.658108000	2.291525000	17.271990000
C	4.969638000	2.948391000	17.580668000
C	3.705297000	2.682999000	18.107781000
C	7.541072000	3.387040000	16.512414000
H	1.386426000	0.676979000	7.507187000
H	-0.671809000	-0.329723000	11.188359000
H	-0.901216000	1.265181000	10.480860000
H	4.973046000	1.393667000	8.534682000
H	6.420675000	-0.430842000	7.812370000
H	5.707098000	-0.936136000	9.367406000
H	3.958999000	-2.257467000	8.116877000
H	4.032695000	-2.508425000	5.697500000
H	2.635591000	-0.801512000	6.593736000
H	2.524059000	0.892868000	4.768259000
H	4.167436000	0.443213000	4.222956000
H	2.822626000	-0.742566000	4.111644000
H	6.049169000	-3.368628000	8.621842000
H	6.649573000	-2.957638000	7.162463000
H	5.621321000	-1.487232000	4.506554000
H	1.668144000	2.063151000	17.676582000
H	5.770873000	3.251111000	18.259577000
H	3.538927000	2.782658000	19.186948000
H	7.388337000	4.339227000	17.056847000
H	8.409125000	3.488293000	15.841661000
H	7.737169000	2.577814000	17.241330000

PMIDA-Dox salt A

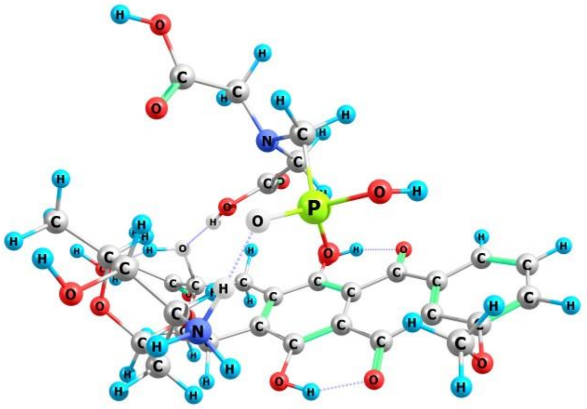

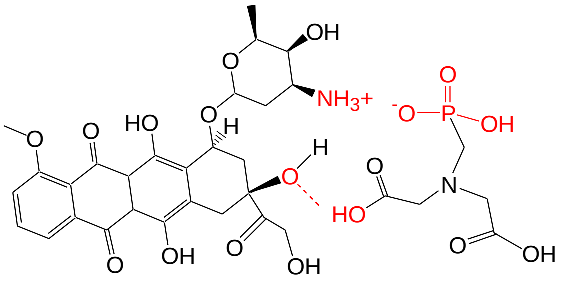

E=-3044.156612084

O	-2.440315000	-0.070689000	14.145249000
H	-3.160904000	-0.681870000	14.378400000
O	-0.025539000	1.877608000	15.819479000
O	0.081233000	-0.675542000	13.403113000
H	-0.883418000	-0.800847000	13.266728000
C	-0.203467000	1.667395000	12.605886000
H	-0.022226000	2.705814000	12.944165000
C	1.811882000	0.894817000	13.907969000
C	2.007790000	1.264531000	11.394234000
C	2.594793000	0.944866000	12.614065000
C	-1.116837000	0.421522000	9.718098000
C	-0.673753000	0.342026000	8.246554000
C	-0.054921000	-1.037095000	7.998992000
C	-1.061015000	-2.150697000	8.344685000
C	-1.565901000	-1.951168000	9.795118000
C	2.805145000	1.351553000	10.207057000
C	4.001058000	0.674647000	12.673014000
C	4.169618000	0.994311000	10.239392000
C	4.767227000	0.639997000	11.488458000
C	-2.705383000	-2.894944000	10.165699000
C	4.944670000	0.987268000	8.987144000
C	6.171987000	0.236520000	11.546885000
C	6.330479000	0.437279000	9.016340000
C	6.925556000	0.101161000	10.266127000
C	7.112123000	0.273870000	7.837438000
P	3.195480000	-3.271715000	7.573051000
C	3.252323000	-4.646654000	8.824968000
H	4.315417000	-4.945733000	8.988395000
H	2.756665000	-5.508921000	8.345366000
N	2.573153000	-4.316921000	10.086465000
C	2.015723000	-5.479648000	10.759732000
C	0.733718000	-6.011320000	10.120481000
O	0.380482000	-7.181468000	10.680751000
H	-0.463004000	-7.484065000	10.284322000
O	0.088587000	-5.472031000	9.246561000
H	2.734523000	-6.328285000	10.854965000
H	1.736413000	-5.215863000	11.799440000
C	3.420546000	-3.517208000	10.967506000
C	2.688574000	-2.733359000	12.054486000
O	1.484757000	-2.305159000	11.682006000
H	1.068559000	-1.710998000	12.370160000
O	3.198209000	-2.480491000	13.131718000
H	3.926320000	-2.751123000	10.348098000
H	4.218067000	-4.112205000	11.469735000
O	1.884040000	-3.377967000	6.772115000
O	3.558996000	-1.931671000	8.203229000
H	0.965367000	-0.409307000	6.265434000
O	4.366251000	-3.765279000	6.499139000
H	5.262183000	-3.585266000	6.839553000
O	0.020935000	0.299313000	10.566482000
O	-2.037338000	-0.601842000	10.023502000
O	-2.106421000	-2.092155000	7.368752000
O	2.208742000	1.770061000	9.083861000
H	2.916110000	1.769193000	8.370323000
O	4.535409000	0.436454000	13.876724000
H	5.499040000	0.213138000	13.727105000
O	4.441494000	1.431572000	7.936882000
O	6.733300000	-0.015669000	12.632134000
O	6.657853000	0.594953000	6.592327000
N	0.409201000	-1.213515000	6.588301000
H	-0.398495000	-1.320248000	5.955584000
C	0.504269000	1.461047000	11.257590000
H	0.323360000	2.360051000	10.634838000
C	0.301715000	0.709561000	13.715892000
C	-0.456237000	1.060624000	15.030792000
C	-1.771708000	0.333273000	15.335289000
H	-2.391426000	1.015479000	15.951893000
H	-1.501861000	-0.536740000	15.976088000
C	8.245144000	-0.370920000	10.334053000
C	8.432241000	-0.202220000	7.930789000
C	8.998720000	-0.519999000	9.167243000
C	5.861342000	-0.399317000	5.923834000
H	-1.293691000	1.562411000	12.465026000
H	2.196665000	0.081526000	14.547326000
H	1.983604000	1.832451000	14.470571000
H	-1.647552000	1.374911000	9.912466000
H	-1.556656000	0.491871000	7.597892000
H	0.054025000	1.143796000	8.037081000
H	0.842184000	-1.160302000	8.628785000
H	-0.539272000	-3.127314000	8.302735000
H	-0.700882000	-2.148423000	10.457431000
H	-3.008266000	-2.736423000	11.216143000
H	-3.585234000	-2.725407000	9.519041000
H	-2.380486000	-3.945048000	10.053407000
H	1.039814000	-2.108880000	6.544122000
H	-2.492757000	-2.977836000	7.268750000
H	8.670557000	-0.617077000	11.311589000
H	9.005227000	-0.312899000	7.002723000
H	10.030378000	-0.887762000	9.219333000
H	6.477367000	-1.287784000	5.675752000
H	5.499133000	0.059529000	4.988470000
H	5.001133000	-0.718331000	6.538015000

PMIDA-Dox salt B

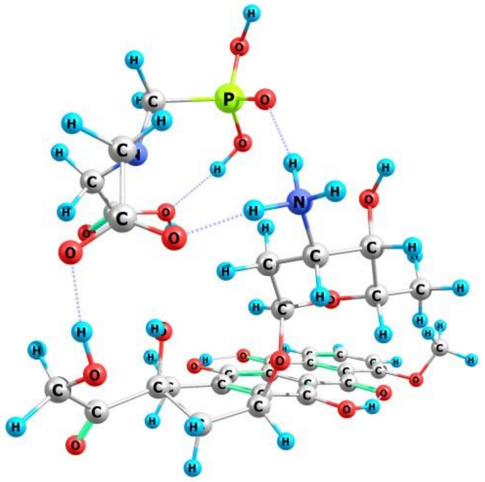

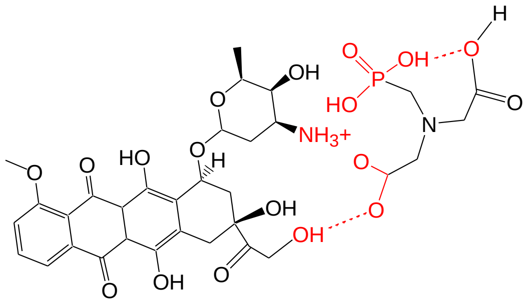

E=-3044.146658006

O	-2.440315000	-0.070689000	14.145249000
H	-3.160904000	-0.681870000	14.378400000
O	-0.025539000	1.877608000	15.819479000
O	0.081233000	-0.675542000	13.403113000
H	-0.883418000	-0.800847000	13.266728000
C	-0.203467000	1.667395000	12.605886000
H	-0.022226000	2.705814000	12.944165000
C	1.811882000	0.894817000	13.907969000
C	2.007790000	1.264531000	11.394234000
C	2.594793000	0.944866000	12.614065000
C	-1.116837000	0.421522000	9.718098000
C	-0.673753000	0.342026000	8.246554000
C	-0.054921000	-1.037095000	7.998992000
C	-1.061015000	-2.150697000	8.344685000
C	-1.565901000	-1.951168000	9.795118000
C	2.805145000	1.351553000	10.207057000
C	4.001058000	0.674647000	12.673014000
C	4.169618000	0.994311000	10.239392000
C	4.767227000	0.639997000	11.488458000
C	-2.705383000	-2.894944000	10.165699000
C	4.944670000	0.987268000	8.987144000
C	6.171987000	0.236520000	11.546885000
C	6.330479000	0.437279000	9.016340000
C	6.925556000	0.101161000	10.266127000
C	7.112123000	0.273870000	7.837438000
P	3.195480000	-3.271715000	7.573051000
C	3.252323000	-4.646654000	8.824968000
H	4.315417000	-4.945733000	8.988395000
H	2.756665000	-5.508921000	8.345366000
N	2.573153000	-4.316921000	10.086465000
C	2.015723000	-5.479648000	10.759732000
C	0.733718000	-6.011320000	10.120481000
O	0.380482000	-7.181468000	10.680751000
H	-0.463004000	-7.484065000	10.284322000
O	0.088587000	-5.472031000	9.246561000
H	2.734523000	-6.328285000	10.854965000
H	1.736413000	-5.215863000	11.799440000
C	3.420546000	-3.517208000	10.967506000
C	2.688574000	-2.733359000	12.054486000
O	1.484757000	-2.305159000	11.682006000
H	1.068559000	-1.710998000	12.370160000
O	3.198209000	-2.480491000	13.131718000
H	3.926320000	-2.751123000	10.348098000
H	4.218067000	-4.112205000	11.469735000

O	1.884040000	-3.377967000	6.772115000
O	3.558996000	-1.931671000	8.203229000
H	0.965367000	-0.409307000	6.265434000
O	4.366251000	-3.765279000	6.499139000
H	5.262183000	-3.585266000	6.839553000
O	0.020935000	0.299313000	10.566482000
O	-2.037338000	-0.601842000	10.023502000
O	-2.106421000	-2.092155000	7.368752000
O	2.208742000	1.770061000	9.083861000
H	2.916110000	1.769193000	8.370323000
O	4.535409000	0.436454000	13.876724000
H	5.499040000	0.213138000	13.727105000
O	4.441494000	1.431572000	7.936882000
O	6.733300000	-0.015669000	12.632134000
O	6.657853000	0.594953000	6.592327000
N	0.409201000	-1.213515000	6.588301000
H	-0.398495000	-1.320248000	5.955584000
C	0.504269000	1.461047000	11.257590000
H	0.323360000	2.360051000	10.634838000
C	0.301715000	0.709561000	13.715892000
C	-0.456237000	1.060624000	15.030792000
C	-1.771708000	0.333273000	15.335289000
H	-2.391426000	1.015479000	15.951893000
H	-1.501861000	-0.536740000	15.976088000
C	8.245144000	-0.370920000	10.334053000
C	8.432241000	-0.202220000	7.930789000
C	8.998720000	-0.519999000	9.167243000
C	5.861342000	-0.399317000	5.923834000
H	-1.293691000	1.562411000	12.465026000
H	2.196665000	0.081526000	14.547326000
H	1.983604000	1.832451000	14.470571000
H	-1.647552000	1.374911000	9.912466000
H	-1.556656000	0.491871000	7.597892000
H	0.054025000	1.143796000	8.037081000
H	0.842184000	-1.160302000	8.628785000
H	-0.539272000	-3.127314000	8.302735000
H	-0.700882000	-2.148423000	10.457431000
H	-3.008266000	-2.736423000	11.216143000
H	-3.585234000	-2.725407000	9.519041000
H	-2.380486000	-3.945048000	10.053407000
H	1.039814000	-2.108880000	6.544122000
H	-2.492757000	-2.977836000	7.268750000
H	8.670557000	-0.617077000	11.311589000
H	9.005227000	-0.312899000	7.002723000
H	10.030378000	-0.887762000	9.219333000
H	6.477367000	-1.287784000	5.675752000
H	5.499133000	0.059529000	4.988470000
H	5.001133000	-0.718331000	6.538015000

SiO_2 Cluster ($Si_{10}H_{18}O_{29}$)

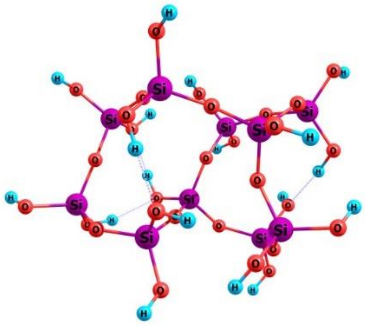

E = -5085.302029576

Si	-0.318690000	-1.001683000	3.992670000
O	-0.639937000	-1.855862000	5.378891000
Si	0.132554000	-3.137809000	6.082642000
O	1.752654000	-2.940516000	5.797000000
Si	3.080434000	-2.231326000	6.486816000
O	4.219703000	-1.984795000	5.313277000
Si	3.683109000	-6.255534000	6.021667000
Si	4.490358000	-1.931237000	3.685517000
Si	3.261806000	-4.693608000	3.195308000
O	4.325843000	-3.442906000	3.026002000
O	3.444528000	-5.302998000	4.705915000
O	3.672737000	-7.842262000	5.581973000
Si	3.356023000	-8.655899000	4.162151000
O	1.879649000	-8.120618000	3.651285000
Si	0.538923000	-7.680553000	2.816770000
O	1.721498000	-4.167605000	2.971493000
Si	0.676216000	-3.344723000	1.986334000
O	0.006324000	-2.074846000	2.775405000
O	-0.385123000	-4.524495000	5.386623000
Si	-0.790015000	-6.117031000	5.220298000
O	-0.358918000	-6.597767000	3.690324000
O	3.367230000	-10.243511000	4.603172000
H	3.309047000	-10.897122000	3.885710000
O	-0.376520000	-8.986790000	2.387776000
H	-0.757079000	-9.518668000	3.107533000
O	1.433044000	-2.698589000	0.665264000
H	1.910126000	-3.296120000	0.064781000
O	3.638793000	-5.833935000	2.040588000
H	2.848175000	-6.249401000	1.619002000
O	3.347582000	-0.876977000	3.074740000
H	3.308676000	-0.786879000	2.104583000
O	5.983563000	-1.344952000	3.323765000
H	6.741370000	-1.941431000	3.453874000
O	-0.206414000	-3.215041000	7.699647000
H	-0.047194000	-2.414829000	8.229190000
O	3.669986000	-3.229643000	7.672746000
H	4.511479000	-3.690537000	7.491986000
O	0.944022000	0.041413000	4.217355000
H	1.804565000	-0.198945000	3.799975000
O	-1.721417000	-0.175260000	3.716935000
H	-1.692085000	0.499674000	3.017213000
O	-0.486578000	-4.428017000	1.477732000
H	-0.955176000	-4.897018000	2.193444000
O	2.669839000	-0.817840000	7.243279000
H	2.183539000	-0.166131000	6.706320000
O	-2.431268000	-6.323924000	5.249525000
H	-2.891152000	-6.025249000	6.052442000
O	-0.069239000	-7.046733000	6.371494000
H	0.800482000	-6.747882000	6.724672000
O	2.455570000	-6.075776000	7.143536000
H	2.498040000	-5.241638000	7.652888000
O	5.134417000	-5.757864000	6.647822000
H	5.470060000	-6.243933000	7.421805000
O	1.068542000	-6.952018000	1.410551000
H	0.477811000	-6.260222000	1.046366000
O	4.502545000	-8.392555000	3.001457000
H	4.453636000	-7.516312000	2.560334000

SiO_2-APS

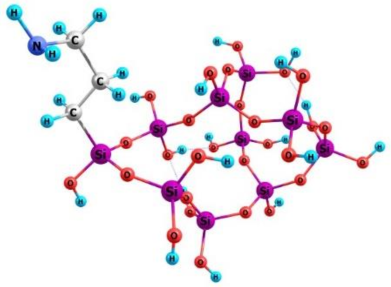

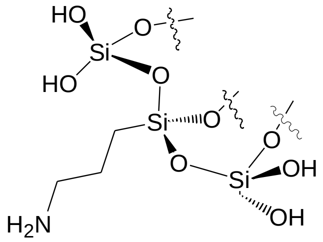

E = -5183.158474308

Si	-0.714376000	1.507781000	5.460559000
O	-0.073815000	0.125515000	6.104052000
Si	-0.363793000	-1.029700000	7.252865000
O	-1.545870000	-0.514943000	8.295565000
Si	-3.186124000	-0.415245000	8.358550000
O	-3.644405000	1.072157000	8.905963000
Si	-1.427140000	0.190387000	12.342931000
Si	-3.614386000	2.709126000	9.060132000
Si	-0.842003000	2.578695000	10.269330000
O	-2.382607000	3.155530000	10.071592000
O	-0.872589000	1.243925000	11.206545000
O	-0.262016000	-0.095372000	13.462984000
Si	1.226360000	0.190013000	14.159286000
C	1.188170000	1.676325000	15.276738000
H	0.373784000	1.543696000	16.018483000
H	2.128071000	1.709239000	15.860238000
C	1.015349000	3.015189000	14.526941000
H	1.806948000	3.108991000	13.754542000
H	0.053563000	3.032351000	13.975695000
C	1.072061000	4.253481000	15.440163000
H	0.834426000	5.150794000	14.821121000
H	0.264660000	4.179602000	16.197415000
N	2.356692000	4.346967000	16.153714000
H	3.100395000	4.567599000	15.480700000
H	2.337367000	5.142998000	16.800902000
O	2.384780000	0.440056000	12.981722000
Si	2.672122000	0.365392000	11.361440000
O	-0.225483000	2.218183000	8.771160000
Si	0.643072000	3.111211000	7.677137000
O	0.044515000	2.809243000	6.168659000
O	1.010461000	-1.303734000	8.112819000
Si	1.307834000	-1.918392000	9.635984000
O	1.605572000	-0.690759000	10.690125000
O	1.624045000	-1.146847000	15.062867000
H	1.570245000	-2.013182000	14.622268000
O	4.215858000	-0.140166000	11.023563000
H	4.321112000	-1.097313000	10.887759000
O	0.015805000	3.829045000	10.948139000
H	0.929794000	3.564910000	11.183571000
O	-3.380647000	3.289905000	7.513421000
H	-3.340833000	4.254219000	7.385351000
O	-5.030602000	3.318082000	9.633329000
H	-5.205249000	3.241951000	10.586883000
O	-0.773290000	-2.450566000	6.509361000
H	-1.569876000	-2.440926000	5.950606000
O	-3.639818000	-1.550443000	9.494052000
H	-4.578823000	-1.574164000	9.751038000
O	-2.356050000	1.541062000	5.707193000
H	-2.743151000	2.285247000	6.231837000
O	-0.356149000	1.460838000	3.849655000
H	-0.765761000	2.146363000	3.295035000
O	2.222111000	2.585592000	7.855604000
H	2.896835000	3.117258000	7.403540000
O	-3.852233000	-0.756255000	6.881547000
H	-3.581874000	-0.114616000	6.190128000
O	2.682362000	-2.837494000	9.564365000
H	2.772087000	-3.414697000	8.787499000
O	0.014261000	-2.781068000	10.174145000
H	-0.657736000	-2.277376000	10.690289000
O	-1.774375000	-1.293291000	11.674529000
H	-2.542068000	-1.372006000	11.061549000
O	-2.761501000	0.913565000	13.015837000
H	-3.212522000	0.400115000	13.707509000
O	2.501246000	1.925943000	10.807661000
H	2.633294000	2.062039000	9.843189000
O	0.493612000	4.745997000	7.899885000
H	0.598740000	5.053568000	8.817753000

SiO_2-2APS

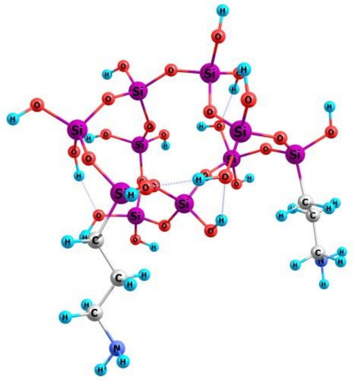

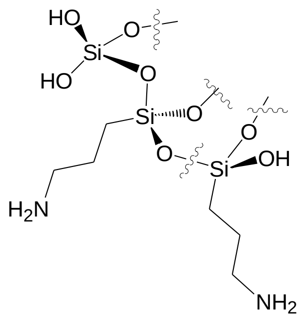

E=-5281.0163333778

Si	-0.332740000	-1.632924000	3.848050000
O	-0.623885000	-2.750694000	5.030770000
Si	0.162306000	-3.467481000	6.295350000
O	1.781107000	-3.479329000	5.953639000
Si	3.081262000	-2.536369000	6.322213000
O	4.127723000	-2.489364000	5.055624000
Si	3.779471000	-6.602018000	5.459800000
Si	4.594604000	-2.269477000	3.492963000
Si	3.238462000	-4.873325000	2.779792000
O	4.431370000	-3.716737000	2.696208000
O	3.284840000	-5.642180000	4.224109000
O	3.224447000	-8.135987000	5.257698000
Si	2.673031000	-9.307815000	4.207159000
C	3.888211000	-9.506350000	2.806949000
H	3.548409000	-10.325897000	2.142110000
H	3.825385000	-8.579703000	2.200592000
C	5.348901000	-9.726070000	3.252162000
H	5.664334000	-8.916096000	3.937093000
H	5.427251000	-10.668624000	3.832420000
C	6.335664000	-9.770326000	2.076266000
H	6.034074000	-10.591777000	1.383516000
H	6.237129000	-8.831507000	1.493771000
N	7.723593000	-9.872397000	2.552954000
H	7.858312000	-10.785460000	3.003312000
H	8.359732000	-9.879656000	1.748618000
O	1.163798000	-8.894292000	3.632917000
Si	0.189942000	-7.643298000	3.177521000
O	1.805869000	-4.073723000	2.606369000
Si	0.800869000	-3.356368000	1.502335000
C	1.659735000	-2.313483000	0.214337000
H	0.868544000	-1.764081000	-0.337927000
H	2.211921000	-1.538624000	0.782529000
C	2.613895000	-3.004293000	-0.781114000
H	3.383032000	-3.599279000	-0.251525000
H	2.047970000	-3.725591000	-1.406135000
C	3.326477000	-1.989153000	-1.689798000
H	2.555948000	-1.364113000	-2.201510000
H	3.908195000	-1.290995000	-1.052635000
N	4.259404000	-2.650677000	-2.612110000
H	3.722888000	-3.193485000	-3.299397000
H	4.752140000	-1.942186000	-3.166323000
O	-0.212809000	-2.369612000	2.376070000
O	-0.415002000	-4.986872000	6.506354000
Si	-0.373180000	-6.546820000	5.961810000
O	0.158513000	-6.517301000	4.395679000
O	2.532369000	-10.740466000	5.040220000
H	1.858956000	-10.778277000	5.741160000
O	-1.358085000	-8.168803000	2.887493000
H	-1.945548000	-8.121729000	3.665337000
O	3.500366000	-5.949247000	1.549910000
H	2.719562000	-6.526199000	1.399153000
O	3.592700000	-1.065205000	2.906381000
H	3.930488000	-0.494434000	2.194138000
O	6.143955000	-1.732110000	3.356389000
H	6.849206000	-2.402221000	3.355778000
O	-0.152964000	-2.705301000	7.733519000
H	0.199463000	-1.804890000	7.840700000
O	3.807397000	-3.313479000	7.600488000
H	4.729557000	-3.083463000	7.810266000
O	1.052329000	-0.775275000	4.207370000
H	1.828440000	-0.791595000	3.601579000
O	-1.680932000	-0.679167000	3.886463000
H	-1.735715000	0.026094000	3.218862000
O	-0.065087000	-4.647752000	0.857328000
H	-0.651163000	-4.483279000	0.097640000
O	2.578199000	-1.019229000	6.765114000
H	2.070537000	-0.567071000	6.058035000
O	-1.898135000	-7.195331000	5.866724000
H	-2.360327000	-7.410577000	6.694529000
O	0.556517000	-7.474481000	6.963758000
H	1.446777000	-7.099288000	7.149113000
O	3.113439000	-6.149770000	6.916439000
H	3.329639000	-5.259123000	7.267809000
O	5.435701000	-6.510667000	5.463748000
H	5.890215000	-7.037062000	6.143907000
O	0.843484000	-7.034308000	1.782923000
H	0.392121000	-6.252671000	1.371575000

SiO_2-PMIDA cluster A

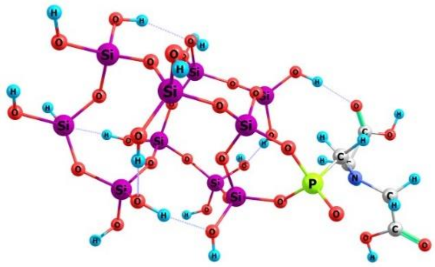

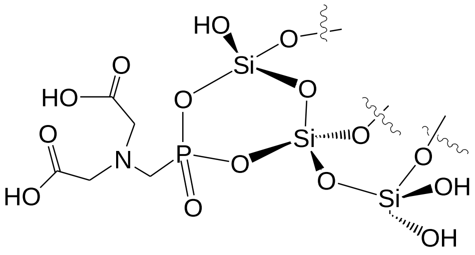

E = -6050.813017988

Si	-0.641400000	-1.609572000	4.544066000
O	-1.315586000	-2.974117000	5.265194000
Si	-0.922365000	-3.921019000	6.556041000
O	0.721338000	-3.986837000	6.732034000
Si	1.705271000	-3.171367000	7.776541000
O	2.935506000	-2.385206000	7.048860000
Si	3.501103000	-6.574508000	6.161987000
Si	3.784639000	-1.817550000	5.743439000
Si	3.195038000	-4.323161000	3.911672000
O	3.662995000	-2.830679000	4.449397000
O	3.036150000	-5.308227000	5.220418000
O	3.478442000	-7.982571000	5.286917000
Si	2.913336000	-8.349098000	3.775219000
O	1.474252000	-7.565634000	3.564292000
Si	-0.125034000	-7.842124000	3.255737000
O	1.744876000	-4.201835000	3.139635000
Si	0.498613000	-3.471733000	2.359571000
O	0.095249000	-2.066782000	3.141457000
O	-1.568721000	-5.402406000	6.400976000
Si	-1.374291000	-7.015929000	6.009258000
O	-1.033595000	-7.051026000	4.391061000
O	2.766972000	-9.982129000	3.586852000
H	1.845231000	-10.307489000	3.579398000
O	-0.253828000	-9.496264000	3.334011000
H	-1.125022000	-9.903534000	3.190423000
O	0.985165000	-3.160739000	0.816987000
H	0.319191000	-2.782092000	0.217287000
O	4.370822000	-4.862246000	2.891789000
H	4.360212000	-5.829001000	2.709046000
O	3.088642000	-0.347362000	5.380865000
H	3.452823000	0.134964000	4.616586000
O	5.353918000	-1.567703000	6.179974000
H	5.980277000	-2.303774000	6.073931000
O	-1.501768000	-3.226139000	7.978166000
P	-0.878683000	-2.048100000	8.934028000
C	-1.560763000	-0.525777000	8.168705000
H	-1.417240000	-0.627770000	7.088331000
H	-2.658788000	-0.576425000	8.340714000
N	-0.944754000	0.709599000	8.628684000
C	-0.585368000	1.656092000	7.569660000
H	-0.031994000	2.503463000	8.004332000
H	0.080486000	1.148544000	6.850567000
C	-1.794950000	2.175978000	6.774650000
O	-2.103261000	3.445246000	7.039046000
H	-2.888011000	3.710090000	6.511073000
O	-2.447907000	1.493007000	5.998518000
C	-1.517565000	1.270239000	9.843706000
H	-2.391979000	1.939020000	9.670174000
H	-1.898970000	0.440574000	10.468700000
C	-0.558434000	2.046411000	10.749984000
O	0.726808000	1.978022000	10.379227000
H	1.263047000	2.504495000	11.008907000
O	-0.943376000	2.654951000	11.727529000
O	-1.200303000	-2.226588000	10.380898000
O	2.292518000	-4.332841000	8.809950000
H	3.021750000	-4.088295000	9.408532000
O	0.437968000	-1.043055000	5.656815000
H	1.275166000	-0.590657000	5.397705000
O	-1.819235000	-0.535477000	4.148693000
H	-2.069653000	0.145594000	4.814984000
O	-0.838082000	-4.475819000	2.319751000
H	-1.418611000	-4.488939000	3.102187000
O	0.724079000	-2.093430000	8.620979000
O	-2.810547000	-7.811010000	6.195625000
H	-3.139938000	-7.907260000	7.105662000
O	-0.238894000	-7.747642000	6.942681000
H	0.668414000	-7.381351000	7.068108000
O	2.391606000	-6.802862000	7.382863000
H	2.341208000	-6.063267000	8.033740000
O	5.023704000	-6.216646000	6.697883000
H	5.436897000	-6.857883000	7.301836000
O	-0.572434000	-7.290313000	1.770112000
H	-0.749882000	-6.323525000	1.745344000
O	4.060915000	-7.735869000	2.727458000
H	3.988980000	-8.017738000	1.797520000

SiO_2-PMIDA cluster B (one free P(O)-OH)

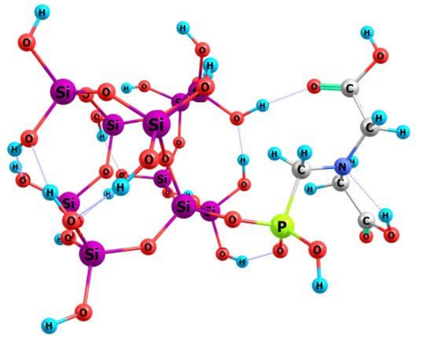

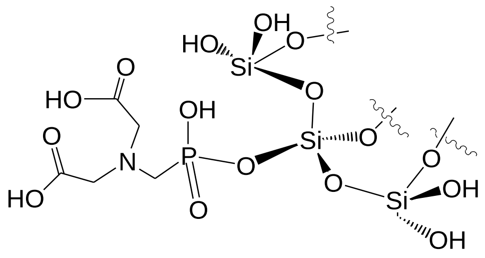

E = -6127.157528097

Si	0.612961000	-1.141127000	3.973450000
O	0.291786000	-2.203881000	5.200200000
Si	0.911958000	-3.477400000	6.028278000
O	2.541983000	-3.409627000	6.102930000
Si	3.826737000	-2.456457000	6.558945000
O	4.837892000	-2.310652000	5.260947000
Si	4.222971000	-6.665376000	5.935317000
Si	5.235278000	-2.464545000	3.671159000
Si	3.679274000	-5.033259000	3.247993000
O	5.010720000	-4.052429000	3.217509000
O	3.566313000	-5.838949000	4.672793000
O	4.541650000	-8.195403000	5.383133000
Si	3.813969000	-9.041524000	4.135821000
O	2.224031000	-8.571807000	4.061415000
Si	0.843057000	-8.064868000	3.312632000
O	2.314706000	-4.115421000	3.081729000
Si	1.500556000	-3.300704000	1.898489000
O	0.786564000	-1.968238000	2.544872000
O	0.362847000	-4.869920000	5.383649000
Si	-0.323606000	-6.349374000	5.728906000
O	-0.137576000	-7.318639000	4.403209000
O	3.964531000	-10.622649000	4.581222000
H	3.726466000	-11.288882000	3.914615000
O	0.037143000	-9.321886000	2.599993000
H	-0.334499000	-9.992809000	3.197963000
O	2.581376000	-2.736679000	0.762161000
H	2.892358000	-3.350214000	0.073563000
O	3.844063000	-6.093018000	1.978124000
H	2.972875000	-6.516185000	1.765522000
O	4.248088000	-1.432614000	2.807473000
H	3.981168000	-1.701600000	1.902698000
O	6.795106000	-2.013968000	3.388766000
H	7.488084000	-2.432948000	3.928004000
O	4.621306000	-3.136072000	7.834407000
H	5.071422000	-3.982536000	7.641234000
O	2.010231000	-0.298202000	4.295699000
H	2.788793000	-0.508098000	3.729520000
O	-0.724903000	-0.182959000	3.936901000
O	5.545533000	-5.908888000	6.593324000
H	6.366711000	-5.860909000	6.072634000
O	1.275052000	-7.024215000	2.083364000
H	0.697722000	-6.261137000	1.854563000
O	4.580888000	-8.797012000	2.691380000
H	4.556920000	-7.882391000	2.335522000
P	-1.174571000	-3.233962000	8.177730000
C	-1.120679000	-4.186167000	9.744287000
H	-0.851541000	-5.231992000	9.477663000
H	-0.281030000	-3.779396000	10.336826000
N	-2.363289000	-4.057842000	10.511947000
C	-3.580375000	-4.623173000	9.910460000
C	-4.619618000	-3.545171000	9.551646000
O	-4.271326000	-2.297123000	9.880597000
H	-3.354597000	-2.346435000	10.252430000
O	-5.686061000	-3.796477000	9.032935000
H	-3.361611000	-5.195930000	8.992283000
H	-4.086951000	-5.330123000	10.594119000
C	-2.233327000	-4.267358000	11.952533000
C	-2.032816000	-5.708586000	12.456946000
O	-1.855451000	-6.597768000	11.468903000
H	-1.738080000	-7.491303000	11.855029000
O	-2.034368000	-6.002386000	13.633563000
H	-3.131666000	-3.876668000	12.466904000
H	-1.386790000	-3.669854000	12.341144000
O	-2.234077000	-3.684122000	7.204213000
O	-1.400435000	-1.676800000	8.551849000
H	-0.722894000	-1.281287000	9.135760000
O	0.339062000	-3.356872000	7.622917000
H	-0.247084000	-4.091815000	0.648936000
O	3.283089000	-0.982589000	7.063182000
H	2.837387000	-0.459335000	6.366538000
O	-1.947373000	-6.187064000	5.961859000
H	-2.207357000	-5.323681000	6.364563000
O	0.364603000	-7.003867000	7.077132000
H	1.347923000	-6.954436000	7.137447000
O	3.095151000	-6.700500000	7.156434000
H	3.433177000	-6.803632000	8.063952000
H	-0.711449000	0.575743000	3.328066000
O	0.381351000	-4.387628000	1.331570000

SiO_2-Dox complex 1

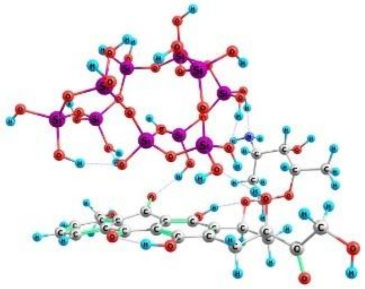

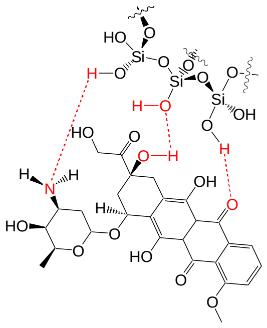

E = -7011.299828736066

O	3.182506000	0.329170000	-0.277129000
O	2.547297000	2.612107000	-0.261390000
O	5.552662000	0.942106000	1.863416000
O	-0.187787000	3.294436000	0.870983000
O	2.988905000	-2.071221000	-1.627522000
O	7.758453000	-3.007402000	0.973831000
O	8.127849000	2.467344000	-0.097497000
O	8.612867000	3.765456000	2.187872000
N	-0.959885000	0.635320000	1.144970000
C	4.494147000	0.180320000	-0.886542000
C	6.376879000	0.994843000	0.699374000
C	5.496424000	1.292853000	-0.529183000
C	7.116424000	-0.344860000	0.479640000
C	4.963371000	-1.231536000	-0.541959000
C	6.180033000	-1.471277000	0.097897000
C	2.309647000	1.338360000	-0.814752000
C	0.855981000	0.895442000	-0.588413000
C	0.451764000	0.975863000	0.895184000
C	0.751725000	2.384775000	1.446392000
C	2.220691000	2.774778000	1.140555000
C	4.125223000	-2.321905000	-0.930570000
C	7.426542000	2.125068000	0.839268000
C	6.580859000	-2.816098000	0.357071000
C	4.499400000	-3.645983000	-0.658215000
C	3.575686000	-4.792136000	-0.911922000
O	2.349089000	-4.649002000	-0.923772000
C	4.190384000	-6.139647000	-1.062898000
C	3.515467000	-7.215357000	-1.713946000
O	2.368745000	-6.943640000	-2.366153000
C	1.690941000	-7.973400000	-3.093066000
H	2.338755000	-8.417699000	-3.873170000
H	1.330476000	-8.773160000	-2.418438000
H	0.827531000	-7.487113000	-3.575008000
C	4.085953000	-8.506355000	-1.681136000
H	3.584148000	-9.345440000	-2.170307000
C	5.760298000	-3.896133000	-0.042011000
C	6.214388000	-5.277269000	0.181105000
O	7.255759000	-5.521408000	0.817870000
C	5.441269000	-6.389931000	-0.443356000
C	5.981828000	-7.682050000	-0.403117000
H	6.934108000	-7.851368000	0.107879000
C	5.294822000	-8.732457000	-1.018041000
H	5.709668000	-9.747229000	-0.992576000
C	2.544162000	4.221590000	1.498998000
C	7.570896000	2.820721000	2.187487000
H	4.347770000	0.222549000	-1.987469000
H	6.172734000	1.433621000	-1.390305000
H	4.951355000	2.241866000	-0.398510000
H	7.690475000	-0.617785000	1.382522000
H	7.868567000	-0.207530000	-0.322432000
H	2.518970000	1.448451000	-1.898177000
H	0.698803000	-0.129797000	-0.971408000
H	0.211681000	1.567979000	-1.184450000
H	1.075385000	0.256304000	1.456225000
H	0.631041000	2.362719000	2.550182000
H	2.870630000	2.101560000	1.736452000
H	5.983129000	0.380716000	2.542513000
H	3.605380000	4.446076000	1.288744000
H	1.918206000	4.915710000	0.911116000
H	2.357436000	4.403936000	2.573373000
H	-1.195271000	-0.257897000	0.698565000
H	-1.560582000	1.345816000	0.712228000
H	7.741784000	2.042896000	2.964486000
H	6.586514000	3.279372000	2.435423000
H	-0.378008000	3.995684000	1.515000000
H	2.604127000	-1.243793000	-1.268155000
H	7.868188000	-3.992582000	1.096321000
H	8.941181000	3.820003000	1.267632000
Si	-0.179909000	-0.974474000	4.153241000
O	-0.666387000	-1.870135000	5.473647000
Si	0.030657000	-3.215934000	6.151703000
O	1.676315000	-3.078239000	6.026782000
Si	2.956353000	-2.192920000	6.601553000
O	4.043649000	-1.930062000	5.378175000
Si	3.692861000	-6.129078000	6.053719000
Si	4.489276000	-1.821682000	3.801189000
Si	3.326162000	-4.572228000	3.219056000
O	4.370676000	-3.299429000	3.072414000
O	3.481837000	-5.187997000	4.731968000
O	3.888720000	-7.706525000	5.623920000
Si	3.481157000	-8.575795000	4.264277000
O	1.951735000	-8.073647000	3.858692000
Si	0.702762000	-7.703223000	2.861001000
O	1.784455000	-4.072552000	2.982816000
Si	0.674696000	-3.275588000	2.038702000
O	0.063747000	-2.008117000	2.888434000
O	-0.441735000	-4.567154000	5.346006000
Si	-0.826078000	-6.158491000	5.129725000
O	-0.303160000	-6.606264000	3.604439000
O	3.539406000	-10.149732000	4.744606000
H	3.445324000	-10.824201000	4.050196000
O	-0.118557000	-9.048492000	2.375753000
H	-0.649240000	-9.518046000	3.041995000
O	1.320303000	-2.622703000	0.676990000
H	1.641916000	-3.249708000	-0.015179000
O	3.756820000	-5.726831000	2.103031000
H	2.977822000	-6.183548000	1.696051000
O	3.481278000	-0.694704000	3.100726000
H	3.776472000	-0.262083000	2.271971000
O	6.037426000	-1.249802000	3.627323000
H	6.768270000	-1.891735000	3.670099000
O	-0.460648000	-3.392121000	7.721039000
H	-0.333411000	-2.637002000	8.320257000
O	3.712764000	-3.007040000	7.825251000
H	4.393165000	-3.663910000	7.570465000
O	1.233598000	-0.197359000	4.582889000
H	2.010934000	-0.264863000	3.977940000
O	-1.358132000	0.102427000	3.787547000
H	-1.303106000	0.393546000	2.810504000
O	-0.506219000	-4.367560000	1.623549000
H	-0.674586000	-5.073131000	2.278395000
O	2.399141000	-0.778632000	7.248721000
H	1.906638000	-0.252341000	6.583597000
O	-2.462944000	-6.390799000	5.071685000
H	-2.962625000	-6.147286000	5.869571000
O	-0.162967000	-7.115300000	6.293185000
H	0.731996000	-6.864573000	6.619604000
O	2.347091000	-6.103993000	7.045645000
H	2.238275000	-5.313338000	7.605278000
O	5.026810000	-5.491300000	6.816551000
H	5.372701000	-5.984099000	7.582872000
O	1.389038000	-7.002903000	1.505386000
H	0.804836000	-6.617719000	0.827627000
O	4.559412000	-8.337178000	3.036785000
H	4.551212000	-7.444285000	2.629874000

SiO_2-Dox complex 2

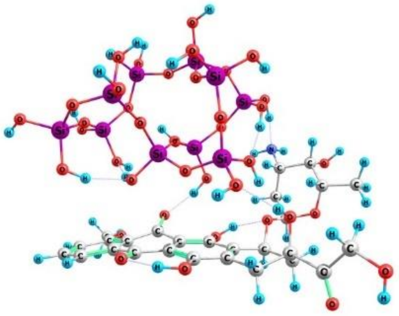

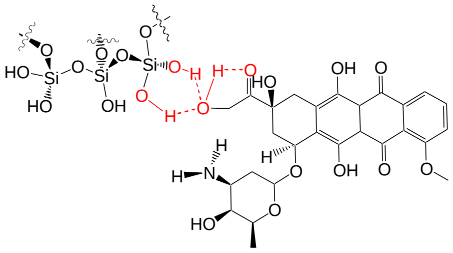

E = -7011.288183504

O	7.581288000	7.539678000	-2.637718000
O	8.019317000	7.905307000	-4.926177000
O	7.685969000	4.737100000	-2.147520000
O	10.883445000	8.357603000	-5.792393000
O	5.933253000	9.833350000	-1.383412000
O	5.255335000	5.522405000	1.966413000
O	4.572942000	3.265003000	-2.933634000
O	5.960144000	1.062723000	-2.913450000
O	5.575559000	11.387369000	0.576839000
O	4.896154000	7.160948000	3.916070000
O	5.571744000	13.005605000	2.637456000
N	11.810044000	8.658643000	-3.247283000
C	6.154371000	7.318043000	-2.503660000
C	6.259428000	4.801305000	-2.137958000
C	5.738358000	5.939876000	-3.034619000
C	5.705886000	4.976581000	-0.692743000
C	5.840974000	7.496792000	-1.028267000
C	5.682024000	6.402120000	-0.188520000
C	8.006034000	8.473888000	-3.634955000
C	9.375951000	9.032075000	-3.241762000
C	10.502585000	8.001954000	-3.412185000
C	10.445110000	7.394141000	-4.841280000
C	9.025093000	6.892647000	-5.166016000
C	5.771216000	8.828953000	-0.513009000
C	5.737620000	3.439091000	-2.617481000
C	5.424465000	6.611458000	1.203315000
C	5.532235000	9.047812000	0.860428000
C	5.357750000	7.922214000	1.723547000
C	8.863126000	6.431603000	-6.612232000
C	6.686613000	2.246950000	-2.598607000
C	5.475701000	10.431604000	1.373192000
C	5.103949000	8.123388000	3.150530000
C	5.314466000	10.639101000	2.834570000
C	5.122088000	9.515188000	3.688377000
C	5.360403000	11.940790000	3.430129000
C	4.962863000	9.671385000	5.071415000
C	5.193771000	12.071569000	4.827200000
C	4.998010000	10.950180000	5.632225000
C	5.712600000	14.312398000	3.207924000
H	5.615758000	8.088758000	-3.083771000
H	4.634703000	5.892809000	-3.066826000
H	6.104197000	5.817048000	-4.069386000
H	6.298446000	4.343735000	-0.007803000
H	4.669101000	4.587216000	-0.645898000
H	7.264923000	9.293662000	-3.684427000
H	9.578683000	9.898580000	-3.898402000
H	9.332645000	9.407666000	-2.202511000
H	10.343468000	7.173846000	-2.687359000
H	11.108828000	6.496843000	-4.869693000
H	8.831672000	6.033598000	-4.489533000
H	8.030640000	5.651391000	-2.181949000
H	7.843361000	6.042667000	-6.786028000
H	9.580792000	5.622400000	-6.836592000
H	9.051753000	7.266453000	-7.309798000
H	12.532524000	7.959422000	-3.044633000
H	11.798518000	9.291792000	-2.441686000
H	7.160416000	2.173950000	-1.598819000
H	7.499873000	2.415222000	-3.330873000
H	11.551451000	8.882186000	-5.297117000
H	5.825584000	10.681011000	-0.853751000
H	5.221658000	13.058352000	5.296127000
H	4.871194000	11.081969000	6.713289000
H	4.779050000	14.644172000	3.701736000
H	5.938251000	14.987476000	2.367334000
H	6.545059000	14.351925000	3.936169000
Si	4.077335000	-7.760528000	-3.126491000
O	4.659275000	-6.499522000	-2.210214000
Si	3.998668000	-5.119976000	-1.583739000
O	2.373441000	-5.110169000	-1.865699000
Si	0.910957000	-5.757681000	-1.441066000
O	0.117589000	-6.165483000	-2.819271000
Si	0.181390000	-2.505249000	-4.042561000
Si	-0.305165000	-7.007019000	-4.163839000
Si	1.040106000	-4.836114000	-5.964147000
O	0.077041000	-6.090648000	-5.487334000
O	1.089188000	-3.652309000	-4.824777000
O	0.557265000	-1.038492000	-4.704295000
Si	1.217893000	-0.570712000	-6.162499000
O	2.828296000	-0.931450000	-6.197514000
Si	4.294492000	-1.161342000	-5.473385000
O	2.560759000	-5.378183000	-6.274051000
Si	3.989246000	-6.032001000	-5.777506000
O	3.701321000	-7.185533000	-4.628911000
O	4.658457000	-3.850409000	-2.438102000
Si	4.401748000	-2.188076000	-2.390597000
O	4.038203000	-1.684227000	-3.917878000
O	0.937830000	1.057721000	-6.233636000
H	1.383867000	1.542325000	-6.948319000
O	5.113203000	0.275648000	-5.514010000
H	5.653688000	0.500877000	-4.727619000
O	4.660511000	-6.686094000	-7.132499000
H	5.559762000	-7.042803000	-7.034625000
O	0.403908000	-4.134173000	-7.332415000
H	0.385868000	-4.657890000	-8.153242000
O	0.553350000	-8.437779000	-4.142432000
H	0.555898000	-8.977197000	-4.953119000
O	-1.908187000	-7.392352000	-4.161813000
H	-2.546633000	-6.663448000	-4.247236000
O	4.389379000	-5.097974000	0.018648000
H	4.078559000	-4.350309000	0.557184000
O	-0.038261000	-4.568387000	-0.759738000
H	0.129192000	-4.332508000	0.170110000
O	2.705360000	-8.354000000	-2.397542000
H	1.968163000	-8.581789000	-3.018111000
O	5.246217000	-8.909498000	-3.321949000
H	5.474379000	-9.441171000	-2.540023000
O	4.987439000	-4.829254000	-5.202435000
H	4.955643000	-4.557016000	-4.259771000
O	1.087054000	-7.009229000	-0.382954000
H	1.674602000	-7.713071000	-0.731771000
O	5.787873000	-1.520990000	-1.803152000
H	5.967334000	-0.589974000	-2.066718000
O	3.167948000	-1.867283000	-1.339715000
H	2.254386000	-2.005413000	-1.684925000
O	0.577030000	-2.382409000	-2.433568000
H	0.197447000	-3.059430000	-1.823494000
O	-1.394489000	-2.963471000	-4.267161000
H	-2.076770000	-2.428545000	-3.826102000
O	5.152861000	-2.251637000	-6.368209000
H	5.113434000	-3.191906000	-6.083269000
O	0.460907000	-1.302260000	-7.434453000
H	0.472887000	-2.283116000	-7.499914000
H	5.070934000	5.844781000	2.894673000
H	4.814823000	8.786330000	5.696298000
H	5.012639000	1.335242000	-2.911332000

SiO$_2$-Dox complex 3

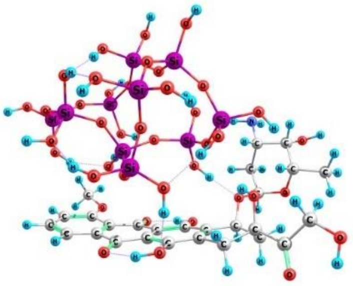

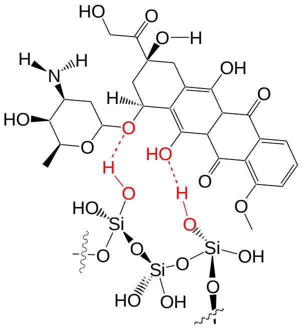

E = -7011.290569604

Si	-2.946915000	-1.502328000	1.711560000
O	-2.010616000	-0.618110000	0.673562000
Si	-0.936003000	0.530602000	0.177835000
O	0.463825000	0.337923000	1.045260000
Si	0.867329000	-0.452706000	2.441190000
O	-0.288583000	-0.088051000	3.565893000
Si	0.990808000	3.437368000	4.024022000
Si	-0.628897000	-0.087636000	5.187047000
Si	-1.955563000	2.467763000	4.419572000
O	-1.612505000	1.226697000	5.465124000
O	-0.541455000	2.926398000	3.682133000
O	1.004142000	5.076390000	4.168128000
Si	-0.150012000	6.278942000	4.242858000
O	-1.110443000	6.143884000	2.909698000
Si	-2.499409000	5.784533000	2.117401000
O	-3.013366000	1.997093000	3.275195000
Si	-4.247130000	1.138777000	2.600930000
O	-3.827271000	-0.473000000	2.652092000
O	-1.587132000	2.036282000	0.423006000
Si	-1.182837000	3.643378000	0.171750000
O	-2.239259000	4.562685000	1.044213000
O	0.588258000	7.746451000	4.125928000
H	1.281905000	7.942621000	4.778594000
O	-3.042868000	7.140413000	1.348005000
H	-3.424663000	7.023667000	0.461429000
O	-5.637516000	1.384925000	3.432680000
H	-6.163051000	0.591078000	3.697165000
O	-2.563864000	3.767073000	5.251087000
H	-3.129223000	4.334796000	4.666155000
O	-1.413057000	-1.530709000	5.417270000
H	-1.800204000	-1.709006000	6.303106000
O	0.742050000	-0.003632000	6.099727000
H	1.138392000	0.896252000	6.098124000
O	-0.628849000	0.451987000	-1.439986000
H	-0.178233000	-0.342574000	-1.774983000
O	2.346240000	0.200693000	2.834138000
H	2.835956000	-0.186868000	3.581062000
O	-2.050016000	-2.506206000	2.701287000
H	-1.869020000	-2.209494000	3.621529000
O	-3.887477000	-2.448799000	0.726170000
H	-4.383719000	-3.162652000	1.160236000
O	-4.453738000	1.658450000	1.040417000
H	-3.615108000	1.876149000	0.588493000
O	0.987875000	-2.089741000	2.254900000
H	0.137999000	-2.572416000	2.293006000
O	-1.481036000	4.074203000	-1.401066000
H	-0.992074000	3.583373000	-2.083788000
O	0.395139000	3.939847000	0.530108000
H	0.898000000	3.479775000	1.235449000
O	1.989629000	3.052710000	2.754026000
H	2.296677000	2.115597000	2.718691000
O	1.434103000	2.689513000	5.446418000
H	2.254978000	3.005596000	5.866034000
O	-3.599607000	5.312357000	3.292441000
H	-4.535416000	5.275569000	3.024908000
O	-1.012793000	6.183234000	5.652421000
H	-1.503779000	5.348290000	5.807694000
O	-7.175639000	-0.837132000	4.073513000
O	-8.659250000	-2.245590000	2.877806000
O	-5.114820000	-3.042744000	4.074492000
O	-9.065437000	-1.779855000	-0.085794000
O	-7.261064000	1.379892000	6.049133000
O	-2.862152000	-1.384951000	7.857407000
O	-5.926710000	-5.506602000	6.539718000
O	-5.279483000	-7.165601000	4.549551000
O	-6.247242000	3.523533000	7.016803000
O	-1.963375000	0.772161000	8.840648000
O	-4.900804000	5.782714000	7.300598000
N	-7.559524000	0.596867000	-0.103036000
C	-7.147180000	-1.341013000	5.431421000
C	-5.609743000	-3.415436000	5.358465000
C	-7.048577000	-2.877574000	5.514130000
C	-4.743614000	-2.828024000	6.481918000
C	-6.022854000	-0.634497000	6.187059000
C	-4.912269000	-1.336470000	6.659346000
C	-8.420599000	-0.939172000	3.365642000
C	-8.432832000	0.084754000	2.220813000
C	-7.514698000	-0.315445000	1.041189000
C	-7.810937000	-1.759920000	0.597266000
C	-7.771464000	-2.700143000	1.825591000
C	-6.143193000	0.767856000	6.455313000
C	-5.627788000	-4.955303000	5.493966000
C	-3.908145000	-0.652259000	7.398403000
C	-5.108209000	1.461831000	7.131283000
C	-3.985334000	0.732913000	7.612443000
C	-8.162099000	-4.139352000	1.503249000
C	-5.255155000	-5.787855000	4.273765000
C	-5.201755000	2.925161000	7.332544000
C	-2.903444000	1.416123000	8.333950000
C	-4.026163000	3.640846000	7.899217000
C	-2.928096000	2.898899000	8.413862000
C	-3.921917000	5.066641000	7.886144000
C	-1.801024000	3.533670000	8.955434000
C	-2.782901000	5.687143000	8.447412000
C	-1.740213000	4.928464000	8.979722000
C	-4.686557000	7.165775000	6.994164000
H	-8.104280000	-1.046710000	5.910341000
H	-7.414146000	-3.192949000	6.508423000
H	-7.724200000	-3.323057000	4.768537000
H	-3.683082000	-3.080021000	6.297429000
H	-4.999480000	-3.332194000	7.434303000
H	-9.247241000	-0.741274000	4.077742000
H	-8.155192000	1.082163000	2.606234000
H	-9.474993000	0.149698000	1.854036000
H	-6.469541000	-0.331846000	1.398938000
H	-6.995886000	-2.074134000	-0.090365000
H	-6.733394000	-2.684942000	2.215220000
H	-4.143015000	-3.037976000	4.100187000
H	-8.090896000	-4.775623000	2.403221000
H	-9.199317000	-4.195685000	1.124164000
H	-7.487341000	-4.556954000	0.733942000
H	-7.207715000	1.516277000	0.180770000
H	-8.531103000	0.734804000	-0.398805000
H	-4.249651000	-5.466623000	3.923512000
H	-5.954414000	-5.510553000	3.452717000
H	-9.129851000	-2.605920000	-0.592032000
H	-7.174983000	2.339674000	6.322135000
H	-2.292181000	-0.755604000	8.399280000
H	-0.976507000	2.925270000	9.336996000
H	-5.624379000	-7.260671000	5.460498000
H	-2.698333000	6.776932000	8.458498000
H	-0.866286000	5.438756000	9.401640000
H	-4.652302000	7.790215000	7.906977000
H	-5.543891000	7.477847000	6.376760000
H	-3.752623000	7.305077000	6.416904000

SiO_2-APS-Dox complex 1

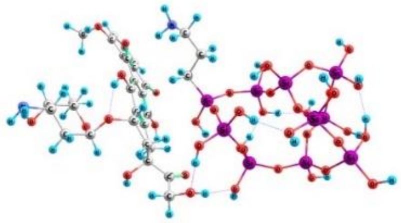

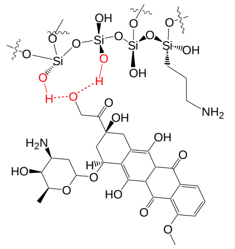

E = -7109.1114435578

Si	-2.606782000	-1.106705000	6.719059000
O	-1.363480000	-1.983779000	7.367046000
Si	-0.838965000	-2.516270000	8.841372000
O	-1.753077000	-1.792162000	10.013325000
Si	-3.205054000	-2.067784000	10.747267000
O	-4.051221000	-0.672309000	10.955118000
Si	-0.510817000	0.170451000	13.183381000
Si	-4.536683000	0.865347000	10.625722000
Si	-1.706092000	1.888772000	10.731874000
O	-3.339045000	1.934662000	11.045292000
O	-0.962906000	0.844634000	11.751307000
O	0.980506000	0.688403000	13.628631000
Si	2.437618000	1.358068000	13.175159000
C	2.731522000	2.875958000	14.215222000
H	2.996922000	2.605616000	15.255036000
H	3.605515000	3.416022000	13.799929000
C	1.479762000	3.792577000	14.184531000
H	1.074685000	3.853210000	13.154203000
H	0.682028000	3.335433000	14.801998000
C	1.728195000	5.228838000	14.666148000
H	0.782544000	5.796221000	14.556878000
O	2.332297000	1.863078000	11.598354000
Si	2.403034000	1.749591000	9.964474000
O	-1.496270000	1.315883000	9.191347000
Si	-1.695558000	1.774091000	7.621743000
O	-2.214233000	0.500121000	6.726957000
O	0.728925000	-2.102413000	9.045156000
Si	1.997783000	-1.383707000	9.807794000
O	1.973521000	0.226470000	9.463882000
O	3.630653000	0.207664000	13.317255000
H	3.468982000	-0.606397000	12.800255000
O	3.917123000	2.119634000	9.412717000
H	4.525519000	1.374695000	9.184302000
O	-1.152255000	3.438497000	10.873050000
H	-0.240008000	3.531547000	10.516005000
O	-4.853423000	0.925017000	8.984827000
H	-4.732123000	1.781962000	8.531160000
O	-5.936032000	1.209337000	11.421110000
H	-5.915556000	1.174937000	12.392986000
O	-0.884608000	-4.168054000	8.926488000
H	-1.742954000	-4.597024000	8.768550000
O	-2.788826000	-2.672007000	12.242305000
H	-3.486852000	-2.721916000	12.919562000
O	-4.004807000	-1.352524000	7.590976000
H	-4.424910000	-0.545035000	7.976540000
O	-2.742858000	-1.650937000	5.167464000
H	-3.463718000	-1.274047000	4.634659000
O	-0.184982000	2.290463000	7.144675000
H	-0.075617000	2.627010000	6.237654000
O	-4.087544000	-3.177675000	9.893633000
H	-4.334774000	-2.851070000	9.002237000
O	3.407328000	-1.961373000	9.126267000
H	3.697280000	-2.847384000	9.407728000
O	2.020606000	-1.694553000	11.434997000
H	1.175682000	-1.768171000	11.942307000
O	-0.338803000	-1.481450000	13.012354000
H	-1.154916000	-1.996865000	12.804749000
O	-1.661391000	0.624444000	14.283886000
H	-1.542214000	0.297151000	15.192104000
O	1.312976000	2.883976000	9.428223000
H	0.939031000	2.752009000	8.524321000
O	7.686652000	3.808289000	12.851336000
O	9.396278000	3.041173000	14.318467000
O	8.537605000	0.426217000	11.783674000
O	11.789808000	4.822189000	14.767528000
O	6.071016000	4.549080000	15.053555000
H	2.455453000	5.713306000	13.970913000
O	6.264074000	-0.865349000	16.187682000
O	5.677086000	-1.444752000	11.393206000
O	5.569731000	-0.104142000	8.848852000
O	5.175792000	4.885070000	17.545291000
O	5.280623000	-0.252905000	18.580283000
O	6.373211000	5.507066000	20.130191000
N	10.827717000	6.866609000	13.039446000
C	6.608080000	2.843548000	12.923284000
C	7.141320000	0.381146000	12.099211000
C	6.548724000	1.780811000	11.813029000
C	6.867950000	-0.104428000	13.543340000
C	6.431763000	2.290031000	14.324200000
C	6.519659000	0.928925000	14.594974000
C	9.059663000	3.400103000	12.993906000
C	9.926081000	4.557879000	12.475537000
C	9.981960000	5.746749000	13.464032000
C	10.421869000	5.236174000	14.849669000
C	9.471563000	4.101954000	15.297808000
C	6.117131000	3.223828000	15.350372000
C	6.464368000	-0.576477000	11.071152000
C	6.226256000	0.460782000	15.911489000
C	5.871378000	2.777350000	16.660355000
C	5.906786000	1.373661000	16.938817000
C	9.836704000	3.451968000	16.628572000
C	6.771247000	-0.351541000	9.577230000
C	5.615995000	3.756698000	17.756039000
C	5.596442000	0.903570000	18.318982000
C	5.884302000	3.292309000	19.150693000
C	5.724569000	1.909463000	19.428543000
C	6.093303000	4.184414000	20.238205000
C	5.674775000	1.438941000	20.746845000
C	6.020102000	3.693291000	21.557942000
C	5.799235000	2.339577000	21.810510000
C	7.292167000	6.004784000	19.146844000
H	5.736271000	3.495837000	12.723589000
H	5.475554000	1.654273000	11.586899000
H	7.004769000	2.198456000	10.898889000
H	7.760454000	-0.673106000	13.879230000
H	6.040210000	-0.835579000	13.498299000
H	9.221467000	2.481134000	12.404525000
H	9.543227000	4.891681000	11.493284000
H	10.950012000	4.168598000	12.318409000
H	8.953924000	6.141940000	13.590593000
H	10.313892000	6.071131000	15.575027000
H	8.475748000	4.572613000	15.411018000
H	8.960909000	-0.401503000	12.073832000
H	9.096748000	2.673961000	16.886221000
H	10.836257000	2.981661000	16.586822000
H	9.836125000	4.207673000	17.435819000
H	10.541196000	7.179594000	12.105982000
H	11.792243000	6.532141000	12.935559000
H	7.292069000	-1.259540000	9.203865000
H	7.446233000	0.502822000	9.415162000
H	12.191942000	4.902423000	15.647516000
H	6.590917000	4.712054000	14.238394000
H	6.557750000	-1.367112000	15.407797000
H	5.531903000	0.368979000	20.926329000
H	4.948082000	-0.863721000	8.950584000
H	6.170423000	4.403952000	22.378760000
H	5.754343000	1.977408000	22.844798000
H	7.930580000	5.195102000	18.746033000
H	7.933954000	6.746563000	19.654420000
H	6.754387000	6.484371000	18.312112000
O	-2.879490000	2.922318000	7.429094000
H	-2.683839000	3.827910000	7.726358000
N	2.137301000	5.285247000	16.077150000
H	2.082534000	6.255113000	16.404887000
H	3.129804000	5.039306000	16.170609000

SiO$_2$-APS-Dox complex 2

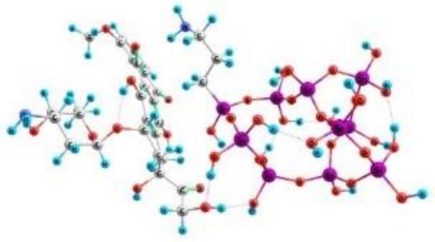

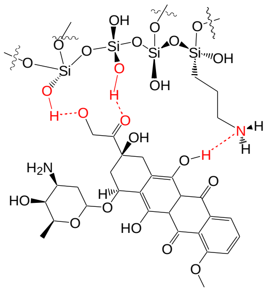

E = -7109.113112645

Si	-1.909516000	-2.976674000	7.788411000
O	-0.831163000	-3.409119000	8.969009000
Si	-0.360919000	-2.957779000	10.483147000
O	-1.625943000	-2.198797000	11.232607000
Si	-2.992764000	-2.535869000	12.087138000
O	-4.298645000	-1.800410000	11.392092000
Si	-0.992512000	1.464018000	13.449593000
Si	-4.873109000	-0.477636000	10.580019000
Si	-2.372194000	1.339738000	10.552485000
O	-3.917401000	0.838856000	10.880939000
O	-1.432168000	1.208211000	11.884618000
O	0.281945000	2.489601000	13.579389000
Si	1.863463000	2.888550000	13.163016000
C	2.360743000	4.558827000	13.857479000
H	1.488404000	4.996844000	14.384138000
H	3.120497000	4.378039000	14.642216000
C	2.882655000	5.569778000	12.806781000
H	3.648305000	5.102294000	12.155420000
H	2.054097000	5.844953000	12.127443000
C	3.472399000	6.860713000	13.406077000
H	3.337093000	7.694453000	12.686891000
H	2.910679000	7.142412000	14.316529000
N	4.905461000	6.718633000	13.771525000
H	5.474868000	6.846126000	12.925950000
H	5.183554000	7.494723000	14.383475000
O	1.905118000	2.962488000	11.495528000
Si	1.683062000	2.299902000	10.009237000
O	-1.749735000	0.328366000	9.397557000
Si	-1.757482000	0.177572000	7.759159000
O	-1.595112000	-1.401522000	7.333648000
O	0.920418000	-1.945814000	10.358808000
Si	1.920862000	-0.764942000	10.920456000
O	1.569591000	0.654221000	10.155736000
O	2.898154000	1.737885000	13.757673000
H	2.742747000	0.823190000	13.439434000
O	2.874629000	2.725649000	8.955036000
H	3.604861000	2.087784000	8.765924000
O	-2.487214000	2.901251000	10.023154000
H	-1.596892000	3.287004000	9.867425000
O	-4.880000000	-0.878564000	8.958831000
H	-4.622126000	-0.190169000	8.304676000
O	-6.431590000	-0.164783000	11.017811000
H	-6.594409000	0.026068000	11.957832000
O	0.152974000	-4.281290000	11.330973000
H	-0.525028000	-4.941406000	11.557881000
O	-2.654328000	-1.959160000	13.612921000
H	-3.385284000	-1.934606000	14.255837000
O	-3.478142000	-3.166760000	8.259612000
H	-4.028830000	-2.378314000	8.480391000
O	-1.540277000	-4.033492000	6.573549000
H	-2.144521000	-4.043673000	5.811703000
O	-0.502704000	1.127939000	7.228360000
H	-0.347925000	1.204215000	6.270280000
O	-3.264415000	-4.167641000	12.166425000
H	-3.826917000	-4.557735000	11.475648000
O	3.456310000	-1.246407000	10.540606000
H	4.158295000	-0.593740000	10.758616000
O	1.821781000	-0.618932000	12.576161000
H	0.960068000	-0.541414000	13.056182000
O	-0.477636000	-0.010024000	14.055200000
H	-1.159115000	-0.722605000	14.016162000
O	-2.325980000	2.073606000	14.213754000
H	-2.202559000	2.380309000	15.128577000
O	0.275275000	2.953709000	9.385083000
H	0.050638000	2.565762000	8.509419000
O	8.457094000	4.568514000	13.334485000
O	12.454354000	3.413075000	15.313894000
O	5.812171000	4.765594000	15.396568000
O	6.944653000	-0.593657000	14.656874000
O	5.364547000	0.749947000	11.100056000
O	4.977185000	1.051482000	8.392422000
O	3.868530000	3.876467000	17.110654000
O	5.493337000	-1.057220000	16.823323000
O	3.910506000	3.864064000	19.942588000
N	11.944682000	6.196762000	15.306893000
C	7.181729000	4.012371000	12.945365000
C	7.424155000	1.996149000	11.233583000
C	7.053814000	3.488871000	11.499010000
C	7.691892000	1.196262000	12.534457000
C	6.654683000	3.004922000	13.955222000
C	6.912744000	1.645698000	13.753701000
C	9.700476000	3.924279000	13.100251000
C	10.793366000	4.958869000	13.404561000
C	10.915493000	5.224019000	14.924528000
C	11.149289000	3.891540000	15.662415000
C	10.033493000	2.884228000	15.297556000
C	5.959445000	3.458537000	15.112528000
C	6.232342000	1.347512000	10.486797000
C	6.558990000	0.704305000	14.755719000
C	5.509870000	2.502823000	16.050741000
C	5.849116000	1.126087000	15.902376000
C	10.276104000	1.477723000	15.845339000
C	6.203186000	1.470825000	8.966582000
C	4.638063000	2.926706000	17.189714000
C	5.471768000	0.161271000	16.971157000
C	4.719783000	2.099029000	18.436255000
C	5.092209000	0.739645000	18.308767000
C	4.346380000	2.587586000	19.716160000
C	5.087286000	-0.113681000	19.422438000
C	4.384533000	1.727934000	20.827354000
C	4.745227000	0.384675000	20.682480000
C	4.876593000	4.922750000	19.905734000
H	6.536075000	4.900035000	12.982031000
H	5.996141000	3.662844000	11.223580000
H	7.646394000	4.117184000	10.810742000
H	8.766223000	1.300340000	12.765640000
H	7.501050000	0.131494000	12.309531000
H	9.757496000	3.569462000	12.051734000
H	10.569810000	5.896881000	12.863372000
H	11.753079000	4.566371000	13.017896000
H	9.940658000	5.612102000	15.281053000
H	11.091751000	4.083151000	16.755239000
H	9.083517000	3.282027000	15.714760000
H	8.942304000	1.094476000	10.337798000
H	9.431836000	0.809644000	15.601415000
H	11.193552000	1.046144000	15.406013000
H	10.390834000	1.498079000	16.944749000
H	11.807766000	7.063327000	14.775641000
H	12.860441000	5.837344000	15.014585000
H	7.063659000	0.898767000	8.562325000
H	6.375282000	2.525004000	8.677516000
H	12.750370000	2.794765000	16.001401000
H	5.470489000	5.389570000	14.660472000
H	7.483497000	-0.726104000	13.858268000
H	5.364951000	-1.163776000	19.287349000
H	4.816245000	0.111810000	8.597894000
H	4.109805000	2.138339000	21.806100000
H	4.763019000	-0.272510000	21.559794000
H	5.714688000	4.715940000	20.600023000
H	4.352967000	5.836301000	20.235360000
H	5.278881000	5.088471000	18.888666000
O	-3.237263000	0.661848000	7.149981000
H	-3.391268000	1.619745000	7.063088000

SiO_2-2APS-Dox complex 1

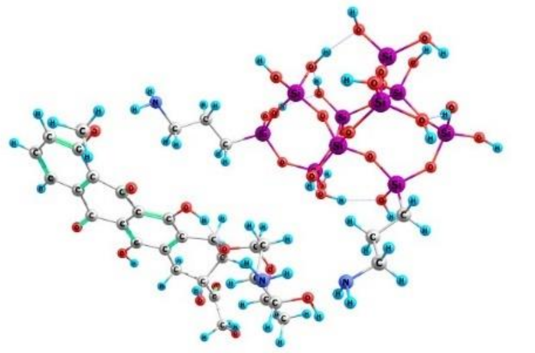

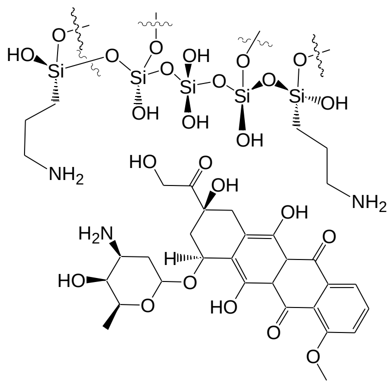

E = -7206.966864318

Si	2.459842000	1.814232000	4.308821000
O	1.657450000	0.548213000	5.020998000
Si	0.426614000	0.344537000	6.107852000
O	0.327631000	1.699363000	7.045591000
Si	-0.553995000	3.089081000	7.026736000
O	0.359770000	4.383453000	7.459230000
Si	0.702783000	1.886043000	10.796460000
Si	1.694359000	5.332650000	7.634763000
Si	3.100784000	3.075909000	9.044667000
O	2.730349000	4.669951000	8.746172000
O	1.767123000	2.348283000	9.641714000
O	1.397912000	0.784647000	11.783079000
Si	2.188567000	-0.548118000	12.386232000
C	2.725241000	-0.184960000	14.131070000
H	3.108042000	-1.109380000	14.609596000
H	3.596167000	0.499077000	14.060953000
C	1.615926000	0.469180000	14.980575000
H	1.120635000	1.276371000	14.405974000
H	0.825387000	-0.277689000	15.202212000
C	2.120132000	1.084266000	16.290972000
H	2.624247000	0.293304000	16.897987000
H	2.907629000	1.824890000	16.054588000
N	1.034860000	1.777746000	16.996953000
H	0.416632000	1.088224000	17.439481000
H	1.431085000	2.329639000	17.764594000
O	3.546965000	-0.841104000	11.452639000
Si	3.975978000	-0.937149000	9.863231000
O	3.506643000	2.355130000	7.620311000
Si	4.691829000	2.134091000	6.483391000
C	5.943156000	3.523676000	6.439797000
H	6.583062000	3.379686000	5.544430000
H	5.365355000	4.451348000	6.240707000
C	6.818632000	3.702336000	7.702041000
H	6.188084000	3.776724000	8.606940000
H	7.456295000	2.807453000	7.852760000
C	7.708309000	4.954025000	7.634043000
H	8.396364000	4.860731000	6.760412000
H	7.067145000	5.834196000	7.418636000
N	8.408612000	5.186283000	8.905686000
H	9.007133000	4.386300000	9.142417000
H	9.035706000	5.993216000	8.825865000
O	3.948596000	2.020209000	5.000659000
O	0.720357000	-0.968399000	7.048014000
Si	1.284846000	-1.566414000	8.479282000
O	2.628640000	-0.712280000	8.916798000
O	1.172888000	-1.867344000	12.322145000
H	0.672994000	-1.954686000	11.481302000
O	4.663197000	-2.399069000	9.483323000
H	4.035411000	-3.061364000	9.137283000
O	4.358165000	3.043816000	10.121057000
H	4.758440000	2.147675000	10.188469000
O	2.379105000	5.373471000	6.116179000
H	3.106794000	5.997688000	5.949751000
O	1.286952000	6.864255000	8.081026000

H	-1.415987000	0.799762000	4.882608000
O	-1.749127000	2.844070000	8.163045000
H	-2.229191000	3.623243000	8.495484000
O	1.570305000	3.217539000	4.441049000
H	1.977099000	3.989893000	4.899849000
O	2.617240000	1.342156000	2.731719000
H	3.121976000	1.937408000	2.151208000
O	5.366723000	0.647633000	6.901272000
H	6.144547000	0.350598000	6.396327000
O	-1.245143000	3.287717000	5.530792000
H	-0.577895000	3.437691000	4.828906000
O	1.809213000	-3.131953000	8.344080000
H	1.148342000	-3.836716000	8.233004000
O	0.089507000	-1.514035000	9.634569000
H	-0.332628000	-0.626326000	9.769259000
O	-0.611418000	1.123464000	10.100271000
H	-1.130580000	1.656065000	9.450957000
O	0.289671000	3.272168000	11.604653000
H	-0.416370000	3.194103000	12.268813000
O	5.094815000	0.267478000	9.630600000
H	5.384832000	0.352001000	8.687103000
O	7.623464000	0.721926000	14.064022000
O	8.612324000	0.192662000	11.977096000
O	10.213246000	-0.412470000	15.397383000
O	9.506844000	2.487177000	10.279609000
O	5.691193000	1.651788000	15.656108000
O	8.069151000	-1.484066000	19.536065000
O	10.110085000	-3.931481000	15.136695000
O	12.530033000	-3.462445000	14.011633000
O	4.856733000	3.190184000	17.676512000
O	6.691066000	-0.168483000	21.324835000
O	2.496426000	3.543922000	19.094979000
N	8.481323000	4.478452000	12.091796000
C	7.280587000	-0.513590000	14.751478000
C	9.472462000	-1.630850000	15.457813000
C	8.271079000	-1.660188000	14.486047000
C	8.922179000	-1.870330000	16.878035000
C	7.124696000	-0.178707000	16.237454000
C	7.801829000	-0.907407000	17.221262000
C	7.497953000	0.760567000	12.620381000
C	7.298127000	2.226316000	12.212169000
C	8.583844000	3.058840000	12.436346000
C	9.746119000	2.376676000	11.688298000
C	9.868511000	0.899995000	12.137197000
C	6.235221000	0.869615000	16.615881000
C	10.440939000	-2.761515000	15.057680000
C	7.465854000	-0.725608000	18.592956000
C	5.909248000	1.069622000	17.973151000
C	6.499692000	0.233391000	18.972683000
C	10.925866000	0.112424000	11.372415000
C	11.832422000	-2.376095000	14.567206000
C	5.010866000	2.199837000	18.379610000
C	6.106213000	0.392214000	20.403655000
C	4.340352000	2.086426000	19.714500000
C	4.910037000	1.250365000	20.702945000
H	10.976539000	-0.929735000	11.733882000

H	1.211719000	7.046866000	9.033246000
O	-1.009030000	0.041565000	5.337739000
C	3.183492000	2.838435000	20.045470000
C	4.374669000	1.206059000	22.000209000
C	2.653127000	2.777708000	21.344058000
C	3.252872000	1.975512000	22.320291000
C	2.657368000	4.967224000	19.047844000
H	6.284611000	-0.839981000	14.377687000
H	7.742631000	-2.618769000	14.633236000
H	8.601368000	-1.626778000	13.436225000
H	9.756490000	-1.730428000	17.593761000
H	8.584399000	-2.922850000	16.961930000
H	6.632646000	0.135168000	12.321055000
H	6.441731000	2.667995000	12.755616000
H	7.039986000	2.240908000	11.138066000
H	8.844453000	3.004228000	13.512699000
H	10.689485000	2.895610000	11.963254000
H	10.140301000	0.896544000	13.210579000
H	10.703243000	0.084446000	10.290094000
H	11.919300000	0.573958000	11.522478000
H	7.751067000	4.912230000	12.665243000
H	8.183098000	4.602863000	11.114520000
H	12.353728000	-1.916741000	15.441972000
H	11.752713000	-1.565410000	13.819526000
H	10.335011000	2.302852000	9.806439000
H	6.268866000	1.581086000	14.861964000
H	8.649291000	-2.145169000	19.119837000
H	4.842983000	0.558637000	22.747850000
H	12.214510000	-4.270598000	14.459864000
H	1.749605000	3.355976000	21.571054000
H	2.825741000	1.936740000	23.329402000
H	2.399859000	5.433761000	20.019065000
H	1.960448000	5.336043000	18.277871000
H	3.690423000	5.234368000	18.760188000
H	9.609247000	0.348946000	15.438524000

SiO_2-2APS-Dox complex 2

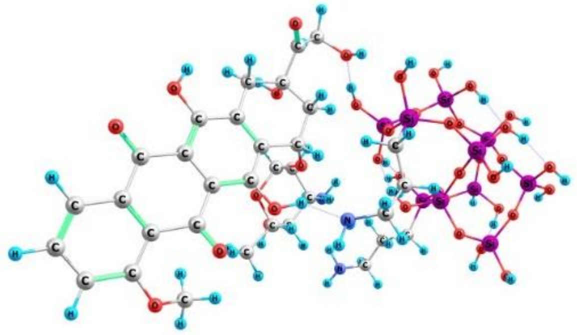

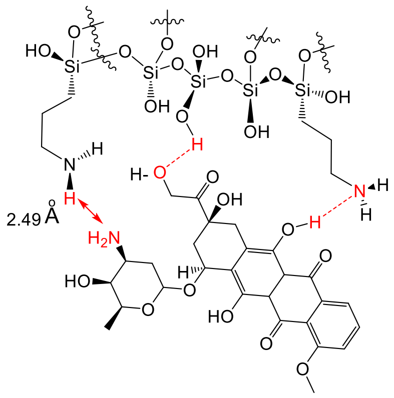

E = -7206.970503408

Si	-1.873393000	2.325998000	5.825989000
O	-1.620642000	0.701087000	5.974632000
Si	-1.648254000	-0.488247000	7.124321000
O	-2.498430000	0.034144000	8.441431000
Si	-3.894787000	0.627359000	9.080160000
O	-3.651920000	2.057515000	9.867209000
Si	-1.143920000	-0.511376000	12.074387000
Si	-3.090182000	3.550261000	10.269822000
Si	-0.395842000	2.309935000	10.671243000
O	-1.630304000	3.362005000	11.042952000
O	-0.792456000	0.778205000	11.116456000
O	0.230944000	-1.289079000	12.543319000
Si	1.810503000	-1.029398000	13.013383000
C	1.842235000	-0.137823000	14.654480000
H	1.430163000	-0.824395000	15.422591000
H	2.898218000	0.016679000	14.933517000
C	1.068925000	1.197325000	14.673337000
H	1.274309000	1.782644000	13.754733000
H	-0.013129000	0.976391000	14.649226000
C	1.337834000	2.106451000	15.881533000
H	0.474177000	2.795526000	15.992650000
H	1.380223000	1.504850000	16.811545000
N	2.600381000	2.873875000	15.748811000
H	2.625074000	3.313939000	14.820107000
H	2.565467000	3.663871000	16.403111000
O	2.588226000	-0.147994000	11.847370000
Si	3.000917000	-0.037701000	10.246763000
O	-0.198328000	2.338011000	9.032467000
Si	0.396719000	3.292542000	7.809818000
C	0.587471000	5.105980000	8.209028000
H	0.880391000	5.585523000	7.249921000
H	-0.409514000	5.520141000	8.458823000
C	1.620131000	5.436647000	9.301500000
H	1.231473000	5.148848000	10.295520000
H	2.535855000	4.833539000	9.155130000
C	2.044122000	6.910793000	9.347699000
H	2.399191000	7.212427000	8.332389000
H	1.164515000	7.552138000	9.567902000
N	3.057338000	7.098126000	10.396876000
H	3.836225000	6.456047000	10.201100000
H	3.449044000	8.044472000	10.347118000
O	-0.615271000	3.154024000	6.503841000
O	-0.107370000	-0.848172000	7.560935000
Si	0.863306000	-1.823586000	8.496990000
O	1.923493000	-0.888216000	9.333689000
O	2.618844000	-2.500876000	13.057039000
H	2.305886000	-3.155815000	13.705177000
O	4.557743000	-0.527482000	10.014071000
H	4.884519000	-1.254928000	10.599420000
O	0.943712000	2.872183000	11.457172000
H	1.783552000	2.514859000	11.084603000
O	-2.960203000	4.363775000	8.815186000
H	-2.893117000	5.334435000	8.829484000
O	-4.111098000	4.425118000	11.223340000
H	-3.183749000	-1.762273000	6.043455000
O	-4.349832000	-0.526437000	10.199934000
H	-5.012470000	-0.269280000	10.865348000
O	-3.316032000	2.735328000	6.555852000
H	-3.264868000	3.440296000	7.246041000
O	-1.913903000	2.588086000	4.195129000
H	-2.164235000	3.481813000	3.905395000
O	1.878949000	2.561091000	7.472090000
H	2.465404000	3.013944000	6.840910000
O	-5.066072000	0.787883000	7.922172000
H	-4.811085000	1.440003000	7.235159000
O	1.816035000	-2.778842000	7.529094000
H	1.346599000	-3.369811000	6.916324000
O	-0.059796000	-2.795854000	9.460857000
H	-0.779701000	-2.418298000	10.017476000
O	-1.967703000	-1.670008000	11.212439000
H	-2.851708000	-1.403108000	10.862476000
O	-2.017130000	0.087129000	13.351314000
H	-2.304535000	-0.561784000	14.016634000
O	2.904988000	1.573998000	9.847148000
H	2.624299000	1.815358000	8.928917000
O	5.355499000	2.012727000	13.740688000
O	6.857425000	3.815948000	13.806729000
O	7.433733000	-0.321137000	12.713631000
O	6.688919000	5.758953000	11.505666000
O	5.143543000	3.073562000	16.678080000
O	9.671936000	-0.059599000	17.093103000
O	6.799421000	-3.325030000	14.504441000
O	5.248813000	-2.605740000	11.668873000
O	5.971925000	4.317951000	18.857745000
O	10.211257000	1.285033000	19.298075000
O	7.499563000	6.697102000	19.466764000
N	4.901603000	4.212552000	10.037616000
C	5.447169000	0.987437000	14.740560000
C	6.930140000	-0.986184000	13.869690000
C	5.518169000	-0.433114000	14.150404000
C	7.841323000	-0.790175000	15.099393000
C	6.485639000	1.258424000	15.816223000
C	7.624799000	0.459898000	15.932371000
C	6.516232000	2.595194000	13.165909000
C	6.235306000	2.812570000	11.672750000
C	5.166245000	3.903082000	11.446446000
C	5.569154000	5.185623000	12.197132000
C	5.893668000	4.878153000	13.678222000
C	6.258691000	2.337987000	16.725503000
C	6.777306000	-2.518216000	13.594000000
C	8.584353000	0.736631000	16.951047000
C	7.244258000	2.654674000	17.691526000
C	8.409657000	1.833649000	17.817756000
C	6.457440000	6.087445000	14.418886000
C	6.531827000	-2.994370000	12.150648000
C	7.066746000	3.826260000	18.599258000
C	9.389922000	2.110539000	18.908885000
C	8.305096000	4.378661000	19.240245000

H	-4.172143000	4.175870000	12.161804000
O	-2.311038000	-1.853552000	6.462124000
C	10.440334000	5.132064000	20.932899000
C	7.082306000	6.920303000	18.112012000
H	4.456751000	1.041439000	15.223675000
H	5.028363000	-1.114595000	14.870784000
H	4.929152000	-0.451318000	13.220572000
H	8.889658000	-0.844935000	14.738161000
H	7.704230000	-1.663062000	15.766612000
H	7.391283000	1.941321000	13.307726000
H	5.914528000	1.856719000	11.220837000
H	7.178285000	3.124555000	11.182573000
H	4.217546000	3.548094000	11.894573000
H	4.706674000	5.883639000	12.174703000
H	4.946236000	4.563832000	14.164624000
H	8.384244000	-0.509806000	12.620359000
H	6.682671000	5.824396000	15.466035000
C	9.365545000	3.481777000	19.520067000
C	8.382740000	5.703109000	19.750707000
C	10.417704000	3.850449000	20.371957000
C	9.443822000	6.054972000	20.607411000
H	7.390638000	6.439300000	13.942943000
H	5.726938000	6.917536000	14.414132000
H	4.559193000	3.371255000	9.565133000
H	5.776060000	4.480817000	9.575469000
H	6.657207000	-4.095310000	12.141105000
H	7.277017000	-2.547336000	11.470947000
H	6.727371000	6.706912000	11.709949000
H	4.359126000	2.701118000	16.173108000
H	9.703442000	-0.719478000	16.379773000
H	11.209175000	3.125769000	20.586678000
H	4.519546000	-2.867708000	12.271500000
H	9.475674000	7.078875000	20.997768000
H	11.256728000	5.429897000	21.601628000
H	7.770624000	6.430468000	17.398376000
H	7.109130000	8.010621000	17.935515000
H	6.059567000	6.540271000	17.945740000

SiO_2-PMIDA-Dox complex 1

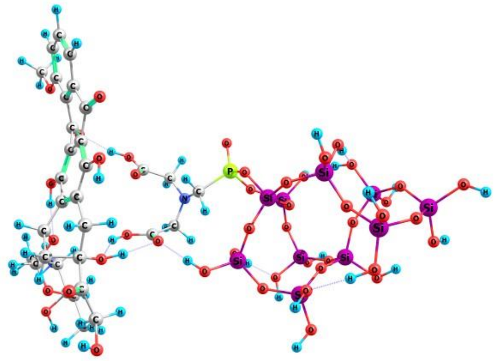

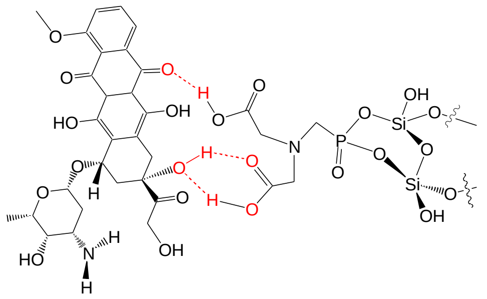

E = -7976.763179977

O	-0.528165000	2.539002000	2.391857000
O	1.728466000	2.462405000	1.710140000
O	0.360498000	0.168893000	4.029166000
O	3.376516000	4.846023000	1.551803000
O	-3.091767000	3.356447000	2.837695000
O	-4.115507000	-1.867822000	4.360587000
O	0.576238000	-2.857510000	2.167748000
O	3.175099000	-2.648702000	2.787629000
O	-5.163177000	3.888241000	4.668528000
O	-6.577481000	-1.066366000	4.973242000
O	-7.626327000	4.692827000	3.776151000
N	1.392668000	6.642923000	2.467250000
C	-1.237810000	1.342005000	1.974438000
C	-0.142938000	-0.707322000	3.023884000
C	-0.331629000	0.119819000	1.736706000
C	-1.513443000	-1.294156000	3.401402000
C	-2.391538000	1.053256000	2.931061000
C	-2.605138000	-0.240553000	3.425744000
C	0.456537000	3.004343000	1.437923000
C	0.474527000	4.538072000	1.385109000
C	1.225531000	5.189878000	2.569846000
C	2.603627000	4.518700000	2.712590000
C	2.428732000	2.993736000	2.857892000
C	-3.339919000	2.077187000	3.208097000
C	0.885091000	-1.824267000	2.735168000
C	-3.867095000	-0.590254000	3.983724000
C	-4.565532000	1.746334000	3.812270000
C	-4.876047000	0.387548000	4.116747000
C	3.751598000	2.243496000	2.975150000
C	2.341873000	-1.562601000	3.104734000
C	-5.536418000	2.801865000	4.220197000
C	-6.262431000	0.034054000	4.532631000
C	-6.975552000	2.442241000	4.144769000
C	-7.324396000	1.085262000	4.331706000
C	-8.003475000	3.417282000	3.991532000
C	-8.669925000	0.694220000	4.384689000
C	-9.350860000	3.004365000	4.034405000
C	-9.671359000	1.656543000	4.231143000
C	-8.610133000	5.722347000	3.646871000
H	-1.713338000	1.583778000	0.997315000
H	-0.806222000	-0.527364000	0.977915000
H	0.638448000	0.441047000	1.328783000
H	-1.447096000	-1.772611000	4.400329000
H	-1.746711000	-2.106980000	2.685686000
H	0.178886000	2.611600000	0.440582000
H	-0.561037000	4.919575000	1.307200000
H	0.989809000	4.820817000	0.447773000

P	-2.277056000	0.501653000	10.935722000
C	-1.585582000	0.810373000	9.265184000
H	-0.710348000	0.154221000	9.186214000
H	-2.348687000	0.390004000	8.575484000
N	-1.188271000	2.169627000	8.957764000
C	0.070501000	2.308845000	8.222277000

H	0.667671000	4.976693000	3.504562000
H	3.094985000	4.908535000	3.628791000
H	1.829799000	2.807822000	3.771549000
H	0.275536000	-0.220307000	4.925682000
H	3.581160000	1.180751000	3.215730000
H	4.321880000	2.304911000	2.030555000
H	4.364788000	2.679229000	3.784780000
H	0.488038000	7.084618000	2.271387000
H	1.980174000	6.846501000	1.650916000
H	2.407475000	-1.312970000	4.184468000
H	2.644665000	-0.633618000	2.569880000
H	4.319226000	4.799249000	1.778159000
H	-2.130828000	3.440076000	2.640685000
H	-3.389061000	-2.453574000	4.085331000
H	-8.918782000	-0.357814000	4.553204000
H	2.634909000	-3.275128000	2.265443000
H	-10.158137000	3.731272000	3.908394000
H	-10.726203000	1.357255000	4.263201000
H	-9.259544000	5.559791000	2.764796000
H	-8.048698000	6.661554000	3.515690000
H	-9.240688000	5.798020000	4.553637000
Si	1.477822000	-1.712567000	9.661525000
O	0.022545000	-2.373746000	10.161591000
Si	-1.096677000	-2.244126000	11.353438000
O	-0.413616000	-1.594727000	12.718888000
Si	-0.413914000	-0.037853000	13.262836000
O	1.103059000	0.497055000	13.545141000
Si	-0.360152000	-3.250216000	16.219972000
Si	2.675542000	0.040818000	13.836409000
Si	2.251526000	-3.043745000	14.404147000
O	2.750103000	-1.462806000	14.506448000
O	0.720596000	-3.171539000	14.983721000
O	-0.769636000	-4.825794000	16.521011000
Si	0.075499000	-6.246843000	16.628421000
O	0.118366000	-7.073176000	15.206668000
Si	0.152628000	-6.831932000	13.564509000
O	2.275025000	-3.457642000	12.810397000
Si	3.030291000	-3.780229000	11.392063000
O	2.721369000	-2.618450000	10.253465000
O	-1.826476000	-3.678219000	11.592438000
Si	-2.100200000	-4.866248000	12.734646000
O	-0.624330000	-5.410654000	13.216814000
O	-0.733427000	-7.109335000	17.779216000
H	-0.447797000	-8.027752000	17.923173000
O	-0.623705000	-8.095198000	12.817498000
H	-1.521859000	-7.880462000	12.498111000
O	4.659199000	-3.845333000	11.658801000
H	5.236113000	-3.777466000	10.878515000
O	3.268844000	-3.980990000	15.305423000
O	-2.879369000	1.936024000	6.632818000
O	-3.611034000	1.101721000	11.235392000
O	-1.324667000	-0.012598000	14.649945000
H	-1.290309000	0.782419000	15.212482000
O	1.555543000	-0.163339000	10.227685000
H	2.261887000	0.042231000	10.885118000

H	0.287888000	3.381342000	8.081612000
H	0.885503000	1.862632000	8.821463000
C	0.098570000	1.596247000	6.859163000
O	-0.011180000	2.397297000	5.807576000
H	-0.031061000	1.870934000	4.965930000
O	0.213760000	0.375113000	6.755676000
C	-2.244026000	3.120899000	8.663725000
H	-3.033823000	3.040597000	9.437588000
H	-1.850082000	4.152279000	8.735237000
C	-2.938661000	2.949312000	7.301933000
O	-3.630734000	4.039082000	6.961942000
H	2.843679000	-4.691069000	15.833212000
O	3.367818000	0.131069000	12.324770000
H	4.306405000	-0.105911000	12.221302000
O	3.433997000	1.120291000	14.817330000
H	3.270499000	1.049920000	15.773517000
O	-2.296254000	-1.132833000	10.932801000
H	-4.067295000	3.925727000	6.073555000
O	1.546777000	-1.759119000	8.019785000
H	1.078705000	-1.025320000	7.561592000
O	2.361725000	-5.230285000	10.906710000
H	2.708846000	-5.638129000	10.094154000
O	-1.129013000	0.879556000	12.044940000
O	-2.841413000	-6.169714000	12.023261000
H	-3.793639000	-6.117913000	11.832805000
O	-3.031235000	-4.297654000	13.960673000
H	-2.672190000	-3.625380000	14.588189000
O	-1.805935000	-2.558214000	15.778597000
H	-1.761061000	-1.622576000	15.468246000
O	0.356742000	-2.511966000	17.516331000
H	-0.195151000	-2.399571000	18.309926000
O	1.743157000	-6.784376000	13.151686000
H	1.964802000	-6.343840000	12.297360000
O	1.676995000	-5.911201000	16.993573000
H	1.858604000	-5.602681000	17.899441000

SiO_2-PMIDA-Dox complex 2

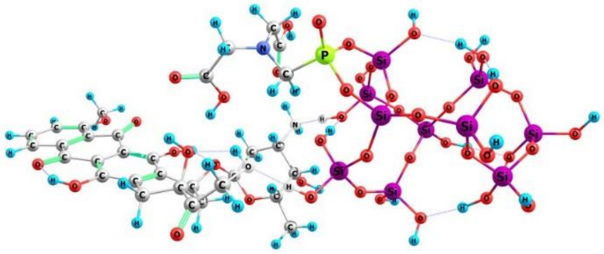

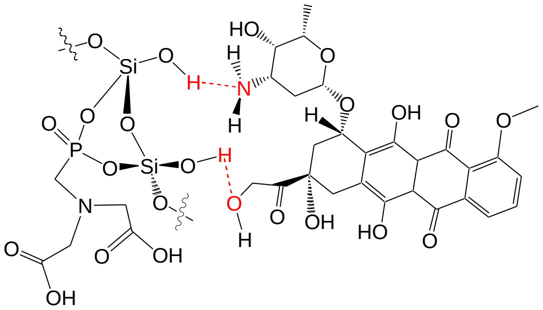

E = -7976.794151513

Si	0.372805000	-1.230571000	4.335369000
O	-0.348825000	-2.744390000	4.348537000
Si	-0.050837000	-4.150340000	5.129321000
O	1.534536000	-4.615996000	5.013388000
Si	2.636541000	-5.094975000	6.144917000
O	4.066554000	-4.340459000	5.938831000
Si	3.315274000	-7.371895000	2.679071000
Si	4.842836000	-3.229964000	4.951029000
Si	3.696924000	-4.270524000	2.216333000
O	4.751903000	-3.780416000	3.387190000
O	3.066436000	-5.744287000	2.610786000
O	3.068192000	-8.042998000	1.185305000
Si	2.302992000	-7.585836000	-0.210822000
O	0.708586000	-7.277504000	0.137810000
Si	-0.186725000	-5.895808000	0.375805000
O	2.452658000	-3.185783000	2.111371000
Si	1.826149000	-1.748957000	1.612972000
O	0.991746000	-0.998320000	2.819387000
O	-1.032047000	-5.336917000	4.572502000
Si	-1.142165000	-6.306217000	3.221555000
O	-0.288959000	-5.571842000	2.002267000
O	2.471997000	-8.764966000	-1.351841000
H	2.250906000	-9.673481000	-1.082851000
O	-1.714628000	-6.089869000	-0.228706000
H	-2.405810000	-6.242404000	0.444446000
O	2.988622000	-0.656099000	1.163904000
H	3.554827000	-0.896869000	0.410640000
O	4.546744000	-4.368257000	0.803382000
H	4.192015000	-5.014200000	0.151766000
O	4.106133000	-1.779783000	5.086126000
H	4.540333000	-0.885029000	5.506468000
O	6.425455000	-3.134644000	5.417530000
H	6.960641000	-3.942186000	5.334886000
O	-0.338066000	-3.949051000	6.772776000
P	0.552713000	-4.060957000	8.138321000
C	0.973894000	-2.322470000	8.583371000
H	1.581011000	-1.889011000	7.765676000
H	0.011706000	-1.779946000	8.558532000
N	1.570011000	-2.209177000	9.896665000
C	2.893431000	-2.719914000	10.151980000
H	3.111479000	-2.630609000	11.232782000
H	2.971587000	-3.806740000	9.932658000
C	4.064276000	-2.053825000	9.415794000
O	3.994330000	-1.139877000	8.625138000
H	5.958877000	-2.219136000	9.275962000
O	5.224442000	-2.651223000	9.758618000
C	0.909949000	-1.399938000	10.888292000
H	-0.174531000	-1.636716000	10.929478000
H	1.293852000	-1.640421000	11.897574000
C	0.985084000	0.128586000	10.736507000
O	1.585218000	0.527421000	9.609791000
H	1.475262000	1.498076000	9.502202000
O	0.493025000	0.889859000	11.549155000
C	4.145419000	2.757528000	6.670602000
C	3.795039000	1.373789000	6.094795000
C	3.245916000	1.561854000	4.668684000
C	1.995047000	2.463035000	4.731749000
C	2.072426000	5.514679000	9.892395000
O	-0.101634000	-4.872982000	9.203242000
O	2.807641000	-6.743800000	6.057947000
H	3.569638000	-7.139617000	6.519583000
O	1.523950000	-1.173037000	5.507773000
H	2.461667000	-1.464902000	5.329702000
O	-0.768659000	-0.075551000	4.583924000
H	-0.932853000	0.182138000	5.524013000
O	0.764297000	-2.154642000	0.382554000
H	0.220130000	-1.441210000	0.004490000
O	1.972957000	-4.706472000	7.635421000
O	-2.711004000	-6.356449000	2.683076000
H	-3.350613000	-6.900426000	3.174520000
O	-0.619500000	-7.830922000	3.547962000
H	0.354902000	-7.987975000	3.570226000
O	2.198110000	-8.077662000	3.697268000
H	2.320916000	-7.782024000	4.631642000
O	4.872290000	-7.602853000	3.184498000
H	5.165653000	-8.525498000	3.281789000
O	0.639471000	-4.745419000	-0.475843000
H	0.595032000	-3.791727000	-0.208644000
O	3.017803000	-6.201940000	-0.817982000
H	2.344288000	-5.503602000	-0.994195000
O	2.101454000	3.517425000	7.710440000
O	2.300370000	3.734488000	5.352490000
O	4.276376000	2.123064000	3.849458000
O	3.292961000	5.245936000	9.369158000
H	3.191205000	4.443487000	8.813845000
O	-1.724108000	6.349354000	11.387327000
H	-1.627917000	6.941181000	12.186374000
O	4.250084000	6.064768000	11.708596000
O	-0.597546000	7.790594000	13.183431000
O	5.396189000	8.055244000	12.982403000
N	4.936860000	0.435580000	6.102036000
C	1.000533000	4.457012000	7.815050000
H	1.238011000	5.323329000	7.164334000
C	-1.243956000	3.359303000	8.403383000
C	-2.511585000	2.701322000	7.796374000
C	-2.515902000	1.173983000	7.596901000
H	-3.245415000	0.943126000	6.801555000
H	-2.914098000	0.733661000	8.538428000
O	-1.271382000	0.581533000	7.234569000
H	-0.612034000	0.830744000	7.912714000
O	-3.499133000	3.354112000	7.525880000
O	-0.522629000	2.373025000	9.159874000
H	-0.889773000	2.287725000	10.060238000
C	-0.312309000	3.839539000	7.276032000
H	-0.859013000	4.587885000	6.676152000
C	-1.606152000	4.572165000	9.271363000
C	0.895736000	5.041213000	9.227429000
C	-0.361039000	5.246475000	9.804523000
C	2.980089000	3.751804000	6.577001000
H	-2.225892000	5.267391000	8.674420000
H	3.360302000	4.790082000	6.666161000
H	4.481752000	2.674610000	7.720403000
C	2.700584000	9.757366000	14.832725000
C	6.573728000	8.649612000	13.532610000
H	-0.058426000	3.009625000	6.600046000
H	-2.245272000	4.261586000	10.119412000
H	4.986612000	3.181793000	6.092183000

C	-0.483038000	6.087639000	10.944782000
C	1.968832000	6.278290000	11.068590000
C	0.673764000	6.623605000	11.560678000
C	1.395629000	2.773860000	3.363281000
C	3.188359000	6.657578000	11.854232000
C	0.525044000	7.524579000	12.710780000
C	3.037163000	7.762166000	12.856338000
C	1.745548000	8.155278000	13.290715000
C	4.175692000	8.409098000	13.428656000
C	1.577394000	9.141768000	14.275479000
C	3.986060000	9.401947000	14.415036000

H	2.999343000	0.904734000	6.701291000
H	2.929890000	0.583989000	4.266243000
H	1.240140000	1.924487000	5.342903000
H	0.495643000	3.405356000	3.466847000
H	2.128126000	3.309234000	2.733164000
H	1.103061000	1.838747000	2.854536000
H	5.221621000	0.226137000	7.065727000
H	5.743464000	0.844238000	5.615611000
H	4.222878000	1.717909000	2.968567000
H	0.568863000	9.420093000	14.595712000
H	4.845824000	9.907039000	14.863388000
H	2.578838000	10.529350000	15.601877000
H	6.608994000	9.740750000	13.346456000
H	7.421718000	8.168675000	13.018771000
H	6.655091000	8.459351000	14.620460000

SiO_2-PMIDA-Dox complex 3

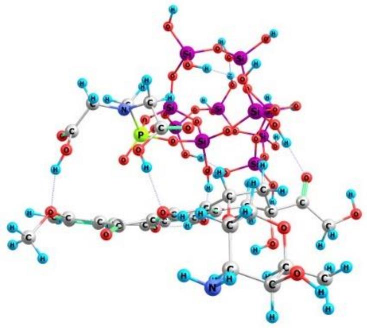

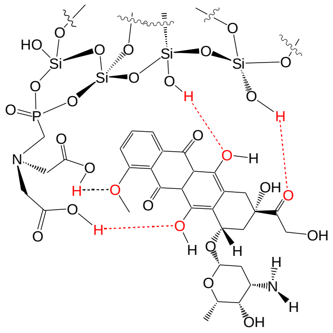

E = -7976.782308837

O	3.732037000	-0.529200000	-2.081363000
O	4.545322000	-1.208484000	0.003611000
O	2.085751000	1.448315000	-0.035878000
O	7.434422000	-1.533537000	0.436358000
O	3.468634000	-0.174291000	-4.708273000
O	-0.834121000	2.957803000	-3.241366000
O	-0.712159000	-0.407822000	1.076746000
O	0.200455000	0.471644000	3.439778000
O	3.598759000	1.324739000	-6.943478000
O	-0.847735000	3.927680000	-5.566365000
O	2.801763000	1.288797000	-9.583621000
N	8.175661000	-0.936614000	-2.222884000
C	2.290312000	-0.703808000	-2.203223000
C	1.033480000	0.530287000	-0.316533000
C	1.546466000	-0.823639000	-0.858885000
C	0.055229000	1.122619000	-1.355254000
C	1.782484000	0.421909000	-3.101668000
C	0.705916000	1.230870000	-2.719771000
C	4.434969000	-1.562723000	-1.352416000
C	5.804532000	-1.820777000	-1.995906000
C	6.819751000	-0.684128000	-1.726776000
C	6.857667000	-0.396494000	-0.213727000
C	5.428993000	-0.098579000	0.293092000
C	2.396416000	0.598123000	-4.378332000
C	0.306521000	0.269445000	1.014386000
C	0.198527000	2.171655000	-3.648080000
C	1.848646000	1.479324000	-5.327887000
C	0.716810000	2.258123000	-4.956545000
C	5.347327000	0.166900000	1.791630000
C	0.889917000	0.860267000	2.281253000
C	2.447179000	1.651509000	-6.694930000
C	0.066008000	3.162966000	-5.926050000
C	1.581135000	2.297475000	-7.737679000
C	0.486795000	3.107983000	-7.345455000
C	1.851141000	2.168344000	-9.120796000
C	-0.255173000	3.831935000	-8.290315000
C	1.077015000	2.864326000	-10.061347000
C	0.047557000	3.715849000	-9.648562000
C	4.021463000	1.846092000	-10.109329000
H	2.117902000	-1.651257000	-2.754125000
H	0.675937000	-1.480239000	-1.022064000
H	2.189764000	-1.315857000	-0.113374000
H	-0.297440000	2.114618000	-1.022986000
H	-0.840017000	0.475983000	-1.423449000
H	3.827427000	-2.488026000	-1.380315000
H	5.685764000	-2.001532000	-3.081195000
H	6.195025000	-2.758007000	-1.556138000
H	6.448734000	0.239255000	-2.215865000
P	-2.461001000	-0.225366000	-6.924249000
C	-2.074471000	-1.909380000	-7.572336000
H	-2.773684000	-2.606425000	-7.067754000
H	-2.371413000	-1.862850000	-8.636790000
N	-0.692810000	-2.325768000	-7.447907000
C	-0.347947000	-3.055034000	-6.240895000
H	-1.183043000	-3.015212000	-5.520919000
H	7.481177000	0.506035000	-0.041310000
H	5.071891000	0.800045000	-0.251417000
H	2.737120000	1.438298000	-0.757356000
H	4.323830000	0.466980000	2.076644000
H	5.624485000	-0.734090000	2.368270000
H	6.032775000	0.987090000	2.070641000
H	8.142302000	-1.189240000	-3.216261000
H	8.553971000	-1.758997000	-1.740562000
H	0.898262000	1.966136000	2.145423000
H	1.957553000	0.557017000	2.333416000
H	7.807774000	-1.251154000	1.287046000
H	3.901703000	-0.462891000	-3.866848000
H	-1.027974000	3.589065000	-4.006271000
H	-1.083437000	4.460410000	-7.950208000
H	-0.602396000	-0.007985000	3.155409000
H	1.288063000	2.716902000	-11.127005000
H	-0.539057000	4.264627000	-10.393973000
H	3.813236000	2.585672000	-10.905365000
H	4.596182000	1.008588000	-10.537713000
H	4.602819000	2.323995000	-9.299792000
Si	-5.531838000	-3.511784000	-5.472314000
O	-5.917160000	-2.032551000	-6.139694000
Si	-5.380464000	-0.484394000	-6.024279000
O	-4.788810000	-0.238098000	-4.494260000
Si	-3.234206000	0.002736000	-3.991267000
O	-2.783136000	-1.028484000	-2.810526000
Si	-5.586110000	2.134470000	-1.169100000
Si	-2.962203000	-2.199619000	-1.654687000
Si	-5.718636000	-1.051390000	-0.945541000
O	-4.279645000	-1.847846000	-0.715178000
O	-5.383508000	0.509998000	-1.345115000
O	-7.122918000	2.484145000	-0.661192000
Si	-8.673385000	2.000737000	-0.940202000
O	-9.026640000	2.130604000	-2.553654000
Si	-9.777787000	1.239324000	-3.736622000
O	-6.503636000	-1.731178000	-2.231701000
Si	-7.253322000	-3.054741000	-2.855569000
O	-6.335458000	-3.738865000	-4.041650000
O	-6.564909000	0.556478000	-6.424676000
Si	-7.573028000	1.784876000	-5.913433000
O	-8.758521000	1.056981000	-5.023374000
O	-9.593996000	3.012755000	-0.015744000
H	-10.554525000	2.862466000	-0.013648000
O	-11.132269000	2.102605000	-4.127016000
H	-11.685335000	1.737367000	-4.839092000
O	-7.628079000	-4.181673000	-1.704858000
H	-6.919160000	-4.761896000	-1.379708000
O	-6.576141000	-1.174570000	0.456331000
H	-7.440752000	-0.708546000	0.408020000
O	-3.169820000	-3.614333000	-2.507765000
H	-3.212424000	-4.449125000	-2.007933000
O	-1.614861000	-2.338668000	-0.730942000
H	-1.392137000	-1.633873000	-0.078549000
O	-4.100690000	-0.206313000	-7.074882000
O	-3.906228000	-3.589713000	-5.190980000
H	-3.610817000	-3.679455000	-4.252986000
O	-6.028100000	-4.588884000	-6.622321000

H	-0.171680000	-4.138971000	-6.437208000
C	0.886897000	-2.567630000	-5.483851000
O	1.701715000	-1.810082000	-6.215423000
H	2.488118000	-1.529159000	-5.688405000
O	1.103775000	-2.893398000	-4.329671000
C	-0.012556000	-2.720790000	-8.681985000
H	0.831871000	-3.383084000	-8.434026000
H	-0.689081000	-3.274047000	-9.369329000
C	0.514449000	-1.488016000	-9.427131000
O	1.822855000	-1.301696000	-9.240587000
H	2.125063000	-0.432424000	-9.607487000
O	-0.189030000	-0.757322000	-10.097824000
O	-1.797422000	0.950795000	-7.555015000
O	-3.147219000	1.580935000	-3.523655000

H	-5.871967000	-5.529591000	-6.430867000
O	-8.671075000	-2.477445000	-3.512760000
H	-9.174561000	-3.103751000	-4.063888000
O	-2.227086000	-0.302838000	-5.300423000
O	-8.293127000	2.481284000	-7.229047000
H	-7.700637000	2.882318000	-7.887861000
O	-6.754609000	2.932774000	-5.081200000
H	-6.428614000	2.827302000	-4.157656000
O	-5.399883000	2.878503000	-2.649573000
H	-4.558799000	2.593720000	-3.086962000
O	-4.476252000	2.622093000	-0.046867000
H	-4.370909000	3.580691000	0.078885000
O	-10.183895000	-0.240515000	-3.127228000
H	-9.610282000	-1.022871000	-3.324557000
O	-8.806081000	0.395995000	-0.475096000
H	-9.437306000	-0.126359000	-1.010922000
H	-2.292456000	2.027933000	-3.305562000

SiO_2-PMIDA-Dox complex 4

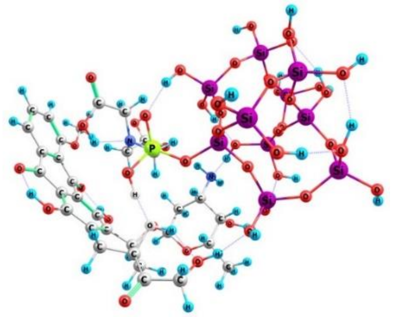

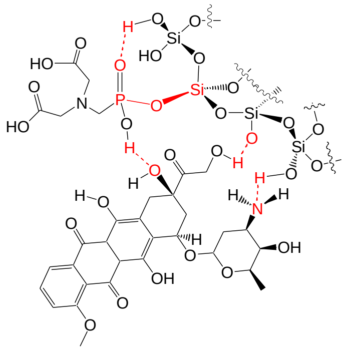

E=-8053.168681839

Si	0.669603000	-0.889006000	3.669005000
O	0.376470000	-1.482802000	5.193781000
Si	0.885427000	-2.661250000	6.220611000
O	2.517683000	-2.723652000	6.212965000
Si	4.072529000	-2.281077000	6.553133000
O	4.905057000	-2.206341000	5.128452000
Si	3.294296000	-6.619150000	6.739000000
Si	5.070484000	-2.863688000	3.623654000
Si	3.150604000	-5.276637000	3.851385000
O	4.568160000	-4.445354000	3.662967000
O	2.921094000	-5.687723000	5.421553000
O	3.453543000	-8.183722000	6.205508000
Si	2.578969000	-9.099883000	5.125101000
O	1.146837000	-8.302433000	4.857741000
Si	-0.083779000	-7.689268000	3.947907000
O	1.885545000	-4.319455000	3.394662000
Si	1.149227000	-3.587694000	2.114665000
O	0.518314000	-2.150039000	2.588866000
O	0.213463000	-4.105360000	5.888116000
Si	-0.833661000	-5.388219000	5.997370000
O	-0.717910000	-6.338334000	4.651559000
O	2.333581000	-10.556238000	5.865632000
H	1.893417000	-11.245938000	5.340091000
O	-1.247775000	-8.831944000	3.674546000
H	-1.680574000	-9.222430000	4.452824000
O	2.282267000	-3.242653000	0.935171000
H	2.511863000	-3.939405000	0.294914000
O	3.235140000	-6.607504000	2.860141000
H	2.345186000	-6.918773000	2.553650000
O	4.130190000	-1.938274000	2.607589000
H	3.748774000	-2.364344000	1.804638000
O	6.628333000	-2.758849000	3.101246000
H	7.303342000	-3.241256000	3.609693000
O	4.759688000	-3.277630000	7.653920000
H	4.810502000	-4.251258000	7.504068000
O	2.153896000	-0.189405000	3.534508000
H	2.906788000	-0.735520000	3.206075000
O	-0.512890000	0.240025000	3.448387000
H	-0.452187000	0.775151000	2.638633000
O	-0.028259000	-4.645770000	1.605884000
H	-0.591662000	-4.382783000	0.855716000
O	4.149624000	-0.775076000	7.264892000
H	4.087067000	-0.004605000	6.672877000
O	-2.388043000	-4.829149000	6.036467000
H	-2.482580000	-3.965481000	6.506053000
O	-0.514109000	-6.267703000	7.356937000
H	0.443599000	-6.409460000	7.582033000
O	3.660489000	-0.181911000	9.923077000
H	3.979764000	-0.375661000	9.009949000
O	2.115726000	2.609328000	11.510469000
O	1.159172000	-0.636720000	10.575818000
H	2.021461000	-0.853873000	10.145840000
C	2.208349000	-0.388660000	12.795110000
H	2.345826000	0.361192000	13.597287000
C	0.076535000	0.772535000	12.159282000
C	-0.040310000	-1.407443000	13.463994000

O	2.150177000	-6.440473000	7.893770000
H	2.402384000	-6.514234000	8.927165000
O	4.738656000	-6.074921000	7.385378000
H	5.563527000	-6.419747000	6.998133000
O	0.547837000	-7.258937000	2.466480000
H	0.176139000	-6.437376000	2.063202000
O	3.428886000	-9.335642000	3.723600000
H	3.675113000	-8.503490000	3.268358000
P	-1.032896000	-2.326173000	8.514020000
C	-0.804276000	-3.748311000	9.670586000
H	-0.391988000	-4.593125000	9.081711000
H	-0.031934000	-3.434463000	10.397656000
N	-2.033737000	-4.097460000	10.401250000
C	-3.171181000	-4.579613000	9.594800000
C	-4.282347000	-3.526244000	9.469040000
O	-4.106058000	-2.445899000	10.242089000
H	-3.218764000	-2.562601000	10.670270000
O	-5.262628000	-3.671745000	8.775443000
H	-2.878023000	-4.911028000	8.583939000
H	-3.642155000	-5.461077000	10.067047000
C	-1.812450000	-4.850290000	11.648428000
C	-1.478938000	-6.335885000	11.435341000
O	-2.559251000	-7.118757000	11.560532000
H	-2.314724000	-8.052423000	11.382580000
O	-0.376318000	-6.760080000	11.155741000
H	-2.720054000	-4.775266000	12.268241000
H	-0.977089000	-4.386933000	12.197729000
O	-2.188144000	-2.485059000	7.560374000
O	-1.149884000	-0.990822000	9.382808000
H	-0.279880000	-0.757197000	9.844085000
O	0.412958000	-2.220175000	7.782958000
O	1.704990000	-2.812622000	12.617957000
O	4.015169000	-3.276351000	12.845230000
O	5.102209000	-5.729432000	11.448896000
O	-0.127310000	-3.295434000	14.902082000
H	-0.810017000	-3.875072000	15.345412000
O	-2.697709000	0.829697000	12.340154000
H	-3.682876000	0.716155000	12.441922000
O	-2.429720000	-4.300698000	15.455614000
O	-4.955926000	-0.332548000	12.890072000
O	-4.363428000	-5.989574000	14.835556000
N	2.579849000	-6.543009000	10.485193000
C	1.469991000	-1.607663000	13.378059000
H	1.854576000	-1.767917000	14.404906000
C	1.432037000	0.289228000	11.641518000
C	2.271284000	1.476194000	11.100654000
C	3.320566000	1.196126000	10.018970000
H	4.204167000	1.824803000	10.253056000
H	2.887884000	1.583310000	9.069357000
C	-6.308746000	-2.792707000	12.980192000
C	-6.296491000	-5.121360000	13.672224000
C	-6.934283000	-4.040862000	13.060062000
C	-4.860298000	-7.326023000	14.710326000
H	3.214465000	-0.702395000	12.475126000
H	-0.518127000	1.193075000	11.329625000
H	0.240715000	1.610082000	12.863642000
H	2.631795000	-3.718144000	14.232855000

C	-0.688255000	-0.334131000	12.850813000	H	3.049111000	-5.822787000	13.132811000
C	2.691169000	-3.704701000	13.126537000	H	1.371155000	-5.383231000	12.738219000
C	2.425404000	-5.108205000	12.564322000	H	2.145142000	-4.497182000	10.503879000
C	2.784555000	-5.202079000	11.068489000	H	4.436525000	-4.677670000	9.773361000
C	4.240421000	-4.749521000	10.859195000	H	3.791320000	-2.651643000	10.866534000
C	4.428620000	-3.337354000	11.460616000	H	5.958004000	-1.823257000	11.789817000
C	-0.798610000	-2.380206000	14.187794000	H	6.534865000	-3.511036000	11.977103000
C	-2.116484000	-0.250835000	12.882154000	H	6.213675000	-2.843114000	10.341086000
C	-2.205323000	-2.381409000	14.097892000	H	1.684279000	-6.925179000	10.809363000
C	-2.867415000	-1.313358000	13.424131000	H	3.316485000	-7.174464000	10.821963000
C	5.870935000	-2.849895000	11.391862000	H	5.910464000	-5.795784000	10.915690000
C	-2.970594000	-3.510360000	14.661882000	H	-6.793839000	-1.948456000	12.481687000
C	-4.327004000	-1.332140000	13.278286000	H	-6.805502000	-6.088169000	13.708474000
C	-4.354448000	-3.707133000	14.159066000	H	-7.930069000	-4.183918000	12.624099000
C	-5.020416000	-2.633778000	13.505478000	H	-5.828710000	-7.453383000	15.230288000
C	-5.013681000	-4.971666000	14.244376000	H	-4.108977000	-7.975397000	15.186254000
				H	-4.967585000	-7.613843000	13.64715100

SiO$_2$-PMIDA-Dox salt 1

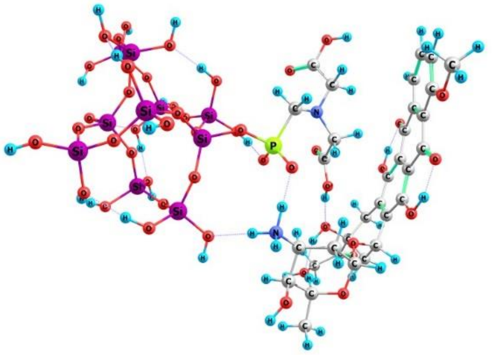

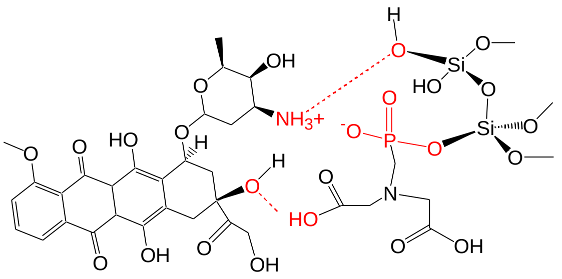

E=-8053.153077257

Si	-1.542743000	-4.664332000	4.313348000
O	0.079993000	-4.429684000	4.193595000
Si	1.624034000	-4.998484000	4.299371000
O	2.219562000	-5.250661000	2.786703000
Si	1.899997000	-5.026438000	1.180879000
O	0.412881000	-5.675764000	0.836354000
Si	3.668993000	-8.832204000	2.250726000
Si	-0.573198000	-6.913950000	0.413410000
Si	0.512621000	-8.869164000	2.472404000
O	0.010192000	-8.367049000	0.980872000
O	2.112732000	-8.523600000	2.670543000
O	4.044579000	-10.423133000	2.471948000
Si	3.526141000	-11.581875000	3.550450000
O	2.976714000	-10.796354000	4.899565000
Si	1.853461000	-10.358942000	6.026241000
O	-0.323439000	-8.030284000	3.611944000
Si	-1.728865000	-7.784083000	4.427151000
O	-1.849523000	-6.205909000	4.857986000
O	1.658232000	-6.375440000	5.183374000
Si	2.527060000	-7.223909000	6.321672000
O	1.922014000	-8.763397000	6.374786000
O	4.857239000	-12.493459000	3.887656000
H	4.760780000	-13.211988000	4.535286000
O	2.131823000	-11.142836000	7.455545000
H	2.100743000	-12.114536000	7.443314000
O	-3.034883000	-8.112586000	3.444540000
H	-3.289101000	-9.045477000	3.325680000
O	0.229128000	-10.498241000	2.606252000
H	0.134681000	-10.769537000	3.556167000
O	-2.058251000	-6.579599000	1.103442000
H	-2.543846000	-7.281669000	1.586203000
O	-0.634572000	-6.935464000	-1.232912000
H	-1.249136000	-7.556377000	-1.660222000
O	3.087072000	-5.690310000	0.255937000
H	3.285373000	-6.651569000	0.302055000
O	-2.321980000	-4.360559000	2.896984000
H	-2.276356000	-5.047052000	2.191974000
O	-2.060425000	-3.565221000	5.450126000
H	-3.021313000	-3.465611000	5.573141000
O	-1.654102000	-8.787955000	5.750165000
H	-2.389949000	-8.798005000	6.387875000
O	1.865688000	-3.393845000	0.925446000
H	1.579862000	-3.086692000	0.048525000
O	2.329351000	-6.566623000	7.812073000
H	2.121081000	-5.585618000	7.773070000
O	4.130543000	-7.236442000	5.918584000
H	4.346623000	-7.475658000	4.989438000
O	-2.989147000	-2.307903000	12.280937000
H	-3.578117000	-2.986286000	11.906465000
O	-1.463240000	-1.589429000	15.379995000
O	-0.299115000	-2.216970000	12.105350000
H	-1.175667000	-2.309874000	11.673296000
C	-0.846338000	0.132590000	12.752406000
H	-0.896387000	0.764142000	13.659993000
H	5.567832000	-7.814077000	3.011396000
O	3.804713000	-8.429864000	0.640872000
H	4.605887000	-8.690937000	0.151438000
O	0.356151000	-10.727976000	5.375756000
H	-0.396417000	-10.171688000	5.690406000
O	2.357806000	-12.528026000	2.865094000
H	1.579933000	-12.046970000	2.515881000
P	2.524579000	-3.113254000	6.471416000
C	4.332965000	-3.153428000	6.881871000
H	4.820313000	-2.359745000	6.263708000
H	4.711009000	-4.124947000	6.513252000
N	4.638622000	-3.028379000	8.305982000
C	6.052667000	-3.078708000	8.607651000
C	6.648264000	-4.492293000	8.617796000
O	7.977085000	-4.543807000	8.854593000
H	8.359734000	-3.654350000	8.987979000
O	6.031270000	-5.516577000	8.442567000
H	6.672194000	-2.458852000	7.912009000
H	6.236891000	-2.663723000	9.617624000
C	3.852891000	-2.064197000	9.063197000
C	2.859130000	-2.710665000	10.039164000
O	1.753818000	-1.971518000	10.135813000
H	1.138618000	-2.250400000	10.862853000
O	3.074466000	-3.717103000	10.684384000
H	3.309190000	-1.362803000	8.406211000
H	4.510855000	-1.430402000	9.685606000
O	1.713727000	-4.008983000	7.412355000
O	2.051354000	-1.667114000	6.285290000
H	0.450244000	-1.313339000	6.279426000
O	2.585740000	-3.815983000	4.966584000
O	-0.069448000	0.280127000	10.427929000
O	-2.076788000	1.205655000	9.627435000
O	-2.936604000	0.332248000	6.889238000
O	2.252730000	2.059978000	10.493295000
H	3.090539000	2.388708000	10.048622000
O	3.614165000	-1.703350000	14.261452000
H	4.603526000	-1.850733000	14.238535000
O	4.697193000	2.220199000	9.818948000
O	6.051640000	-1.539153000	13.475474000
O	6.980846000	1.821443000	8.569902000
N	-0.605620000	-1.132114000	6.376141000
H	-0.903142000	-0.433862000	5.678336000
C	0.154738000	0.756476000	11.765277000
H	0.012446000	1.855910000	11.784527000
C	-0.445200000	-1.298141000	13.199600000
C	-1.531253000	-1.812078000	14.187493000
C	-2.705060000	-2.631604000	13.637769000
H	-3.575065000	-2.451387000	14.301249000
H	-2.415648000	-3.700368000	13.755937000
C	7.939318000	-0.958041000	11.531909000
C	8.556966000	0.273150000	9.536450000
C	8.867163000	-0.652494000	10.533201000
C	7.886317000	2.098201000	7.497050000
H	-1.850347000	0.146770000	12.296732000
H	1.267725000	-2.253877000	14.113459000
H	0.795776000	-0.732664000	14.878589000

C	0.917377000	-1.229631000	13.897537000	H	-0.317775000	2.184148000	9.646283000
C	1.610308000	0.447549000	12.111665000	H	-0.561653000	1.423140000	7.350800000
C	1.953263000	-0.497815000	13.073235000	H	0.840893000	0.555743000	8.039824000
C	-0.664980000	1.144603000	9.487723000	H	-0.585957000	-1.464837000	8.440479000
C	-0.257044000	0.655862000	8.086582000	H	-2.937932000	-1.544330000	7.822942000
C	-0.949622000	-0.675192000	7.760347000	H	-2.456980000	-0.804829000	10.053888000
C	-2.474577000	-0.539175000	7.921314000	H	-4.444173000	0.589106000	10.611846000
C	-2.786847000	-0.017389000	9.345691000	H	-4.633790000	1.045577000	8.889261000
C	2.631741000	1.100424000	11.347694000	H	-4.861707000	-0.660605000	9.400981000
C	3.329519000	-0.827389000	13.287951000	H	-1.094701000	-2.020021000	6.129243000
C	3.979818000	0.701265000	11.475296000	H	-3.864946000	0.125337000	6.691774000
C	4.327023000	-0.277999000	12.455764000	H	8.169514000	-1.691210000	12.310071000
C	-4.269891000	0.257152000	9.572949000	H	9.298825000	0.486720000	8.762725000
C	4.995424000	1.266601000	10.565271000	H	9.847046000	-1.144647000	10.523139000
C	5.704982000	-0.753468000	12.570763000	H	8.832454000	2.538850000	7.866430000
C	6.341300000	0.631480000	10.537091000	H	7.374969000	2.829584000	6.850995000
C	6.685388000	-0.332114000	11.528003000	H	8.109946000	1.186723000	6.909859000
C	7.305641000	0.930240000	9.521484000				
O	4.616267000	-7.861644000	3.216716000				

SiO_2-PMIDA-Dox salt 2

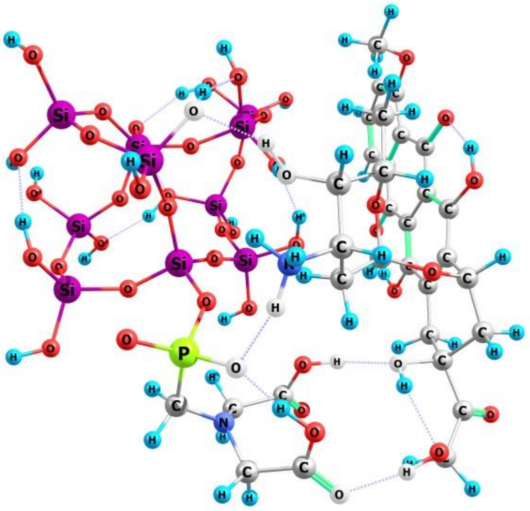

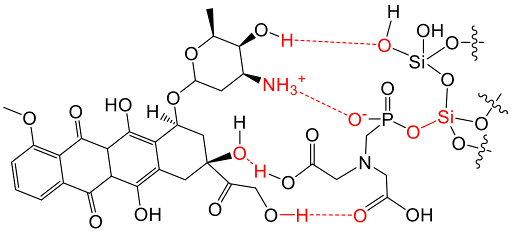

***E*=-8053.169980512**

Si	0.383497000	-7.245139000	4.852072000
O	0.605149000	-6.503038000	6.304523000
Si	1.350628000	-5.332394000	7.184649000
O	2.589620000	-4.650102000	6.339695000
Si	3.280539000	-3.645252000	5.234858000
O	3.827357000	-4.552881000	3.964880000
Si	5.732348000	-5.030827000	8.442490000
Si	4.681156000	-5.877216000	3.495532000
Si	5.069345000	-7.287126000	6.219051000
O	5.467130000	-6.582523000	4.779539000
O	5.132044000	-6.182707000	7.424920000
O	6.713400000	-5.767026000	9.538764000
Si	7.308206000	-7.299837000	9.796785000
O	6.059956000	-8.321779000	10.159448000
Si	4.580302000	-8.991166000	9.880460000
O	3.532711000	-7.890200000	6.131722000
Si	2.741584000	-9.148997000	5.405476000
O	1.256909000	-8.669261000	4.900459000
O	1.901061000	-5.981620000	8.572271000
Si	2.293701000	-6.699786000	9.999332000
O	3.429226000	-7.851192000	9.654246000
O	8.317830000	-7.123436000	11.086081000
H	8.632090000	-7.921300000	11.542614000
O	4.104253000	-9.904747000	11.172124000
H	4.682631000	-10.638785000	11.440738000
O	3.536243000	-9.622263000	4.018219000
H	4.349581000	-10.151038000	4.105470000
O	6.162192000	-8.511463000	6.492596000
H	5.817310000	-9.185606000	7.132220000
O	3.569066000	-6.943055000	2.850417000
H	3.729296000	-7.908083000	2.941354000
O	5.777925000	-5.328318000	2.395442000
H	6.319443000	-5.983629000	1.923234000
O	4.534805000	-2.767126000	5.877789000
H	5.345569000	-3.194102000	6.237543000
O	0.857995000	-6.337212000	3.560166000
H	1.756207000	-6.510093000	3.197088000
O	-1.233443000	-7.570214000	4.778757000
H	-1.590970000	-7.854094000	3.920318000
O	2.681036000	-10.329260000	6.575127000
H	2.236856000	-11.173535000	6.378660000
O	2.133583000	-2.564640000	4.744486000
H	2.417127000	-1.832658000	4.170029000
O	0.986034000	-7.407274000	10.730168000
H	0.630195000	-8.211270000	10.315497000
O	2.850601000	-5.594169000	11.092451000
H	3.506512000	-4.977987000	10.695121000
O	-2.922605000	-1.414072000	13.328648000
C	2.851425000	-4.609511000	14.826468000
C	4.838735000	-3.306292000	14.191343000
C	4.239331000	-4.542130000	14.589123000
C	5.270140000	-0.840102000	10.244652000
C	6.282785000	-3.204155000	13.935982000
C	5.064300000	-5.734677000	14.791639000
C	7.106124000	-4.445232000	14.005073000
C	6.523026000	-5.646384000	14.502110000
C	8.490646000	-4.464528000	13.673627000
O	4.555931000	-4.261856000	9.293840000
H	4.078327000	-3.466811000	8.939348000
O	6.538158000	-3.881964000	7.536164000
H	7.376740000	-4.140393000	7.111838000
O	4.769477000	-9.888667000	8.482114000
H	3.960238000	-10.192467000	8.008982000
O	8.167289000	-7.829280000	8.488977000
H	7.660336000	-8.081061000	7.687500000
P	-1.228270000	-3.705419000	6.917731000
C	-2.433388000	-4.986491000	7.504507000
H	-2.040327000	-5.974144000	7.198682000
H	-3.353785000	-4.827523000	6.904169000
N	-2.715351000	-4.947784000	8.930571000
C	-3.898651000	-4.242766000	9.386687000
C	-3.624859000	-3.021944000	10.293536000
O	-2.778929000	-2.109017000	9.871944000
H	-2.317279000	-2.307373000	8.967433000
O	-4.210532000	-2.889939000	11.367139000
H	-4.468630000	-3.863673000	8.514643000
H	-4.581104000	-4.909724000	9.953371000
C	-2.259227000	-6.039563000	9.770874000
C	-1.723670000	-5.584318000	11.136114000
O	-0.885051000	-4.555245000	11.026495000
H	-0.683059000	-4.148292000	11.913847000
O	-2.036065000	-6.099652000	12.193264000
H	-1.422424000	-6.562543000	9.271897000
H	-3.052722000	-6.793887000	9.973295000
O	-1.151992000	-3.691628000	5.409217000
O	-1.533580000	-2.368337000	7.658478000
H	-0.160879000	-1.348922000	7.927641000
O	0.225885000	-4.179659000	7.583109000
O	1.575901000	-0.935935000	12.320810000
O	3.323784000	-1.945721000	11.123253000
O	3.347463000	-1.982054000	8.251383000
O	4.529832000	-0.956717000	13.763888000
H	5.524368000	-1.075344000	13.698030000
O	2.249424000	-5.725956000	15.256796000
H	2.961367000	-6.424149000	15.343803000
O	6.797180000	-2.095425000	13.678471000
O	4.568917000	-6.808668000	15.188038000
O	9.146631000	-3.397572000	13.132001000

H	-3.486011000	-1.784360000	12.602450000
O	-1.640371000	-2.479387000	16.403147000
O	-0.633480000	-2.794537000	12.994983000
H	-1.297375000	-2.141904000	12.676174000
C	0.367418000	-1.110167000	14.459020000
H	0.516888000	-0.866663000	15.528206000
C	0.549508000	-3.600812000	14.907042000
C	2.599133000	-2.275414000	14.155116000
C	2.023079000	-3.463788000	14.594336000
C	1.937639000	-1.983453000	11.442648000
C	1.075376000	-1.920542000	10.167602000
C	1.503531000	-0.783069000	9.231106000
C	3.030889000	-0.800827000	8.999499000
C	3.749026000	-0.801828000	10.364677000
C	4.022378000	-2.164050000	14.029994000
H	5.725747000	-0.867202000	11.247915000
H	5.613026000	-1.726909000	9.685939000
H	5.631241000	0.063567000	9.720956000
H	0.711355000	0.022452000	7.438143000
H	3.886448000	-1.796107000	7.453243000
H	6.821125000	-7.694382000	15.109405000
H	10.322676000	-5.588578000	13.670079000
H	9.287300000	-7.657935000	14.639263000
H	9.227202000	-4.027087000	11.130119000
N	0.803728000	-0.894988000	7.898599000
H	1.358643000	-1.496693000	7.265116000
C	1.736692000	-1.084696000	13.763242000
H	2.252815000	-0.162642000	14.084460000
C	-0.346975000	-2.479833000	14.359334000
C	-1.665655000	-2.414338000	15.189783000
C	-2.999800000	-2.294988000	14.441874000
H	-3.761413000	-1.967697000	15.179131000
H	-3.266999000	-3.329581000	14.129302000
C	7.302809000	-6.791397000	14.722347000
C	9.256292000	-5.617009000	13.921817000
C	8.673581000	-6.768230000	14.454531000
C	8.913616000	-3.153559000	11.734628000
H	-0.270704000	-0.315804000	14.030291000
H	0.167497000	-4.571268000	14.541749000
H	0.444797000	-3.629004000	16.009043000
H	1.791504000	-2.968407000	11.913691000
H	1.186871000	-2.888055000	9.648838000
H	0.013178000	-1.819939000	10.441692000
H	1.217021000	0.192935000	9.659161000
H	3.333137000	0.100059000	8.430020000
H	3.453687000	0.126420000	10.897273000
H	9.525115000	-2.278708000	11.458062000
H	7.851143000	-2.931474000	11.524417000

SiO_2-PMIDA-Dox salt 3

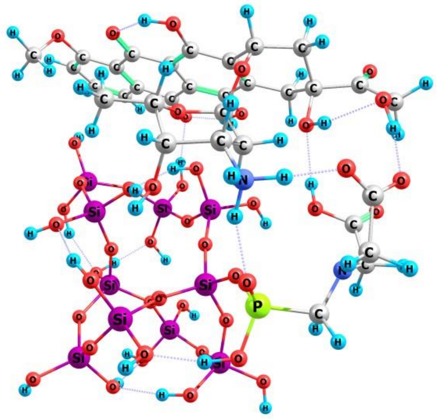

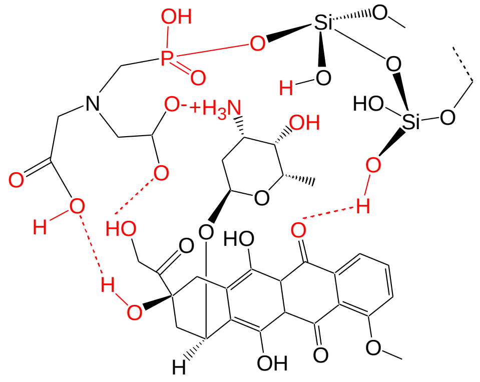

E= -8053.168327294

Si	4.070740000	-5.156226000	6.316634000
O	4.137348000	-4.139568000	7.618322000
Si	4.891449000	-2.865781000	8.320246000
O	6.075858000	-2.277269000	7.342952000
Si	6.732878000	-1.367856000	6.137756000
O	7.663275000	-2.334523000	5.181772000
Si	9.199391000	-1.994977000	9.421734000
Si	8.458098000	-3.734429000	4.855762000
Si	8.754397000	-4.714288000	7.741468000
O	9.226945000	-4.288813000	6.219474000
O	8.681769000	-3.384092000	8.693669000
O	10.341121000	-2.434750000	10.526999000
Si	10.762656000	-3.891995000	11.218115000
O	9.368625000	-4.684237000	11.626306000
Si	8.171966000	-5.817779000	11.620225000
O	7.243351000	-5.389323000	7.703965000
Si	6.544432000	-6.792495000	7.172098000
O	5.007106000	-6.487499000	6.688687000
O	5.446181000	-3.264878000	9.790540000
Si	5.791887000	-3.751908000	11.334667000
O	6.721592000	-5.121924000	11.243616000
O	11.649453000	-3.459758000	12.537883000
H	11.987783000	-4.170036000	13.108338000
O	8.129924000	-6.625230000	13.055071000
H	8.088885000	-6.091613000	13.878668000
O	7.335848000	-7.361817000	5.819926000
H	8.165062000	-7.859335000	5.937421000
O	9.865929000	-5.813265000	8.302482000
H	9.515666000	-6.373838000	9.044153000
O	7.328488000	-4.856969000	4.349704000
H	7.492987000	-5.802866000	4.564576000
O	9.561878000	-3.360537000	3.693949000
H	10.037075000	-4.090772000	3.261895000
O	7.589660000	-0.081467000	6.702893000
H	8.445317000	-0.256714000	7.159052000
O	4.577713000	-4.461615000	4.912365000
H	5.512564000	-4.579010000	4.625296000
O	2.480116000	-5.578119000	6.213563000
H	2.202770000	-6.080781000	5.428873000
O	6.618141000	-7.829027000	8.467613000
H	6.220244000	-8.715467000	8.393893000
O	5.452147000	-0.764888000	5.253227000
H	5.621093000	-0.043389000	4.620268000
O	4.411498000	-4.078685000	12.187490000
H	3.907604000	-4.874343000	11.947255000
O	6.556568000	-2.563088000	12.171571000
H	7.093038000	-1.941367000	11.625904000
O	0.729841000	0.764336000	14.236244000
H	0.269653000	0.728590000	13.338534000
O	1.303321000	-1.431451000	16.952668000
O	2.846066000	-0.854157000	13.849425000
H	2.260934000	-0.105132000	13.591518000

N	0.694037000	-0.948694000	9.284107000
C	0.177161000	0.420782000	9.422539000
C	0.433118000	1.017830000	10.832619000
O	1.297280000	1.928238000	10.957661000
O	-0.261911000	0.537553000	11.774967000
H	0.678821000	1.055068000	8.675267000
H	-0.921691000	0.457383000	9.222403000
C	0.030634000	-1.972467000	10.076480000
C	0.633310000	-2.237554000	11.460178000
O	1.938227000	-1.965694000	11.526158000
H	2.264274000	-2.027081000	12.457867000
O	-0.008434000	-2.714679000	12.378435000
H	0.077555000	-2.943397000	9.544018000
H	-1.046469000	-1.758288000	10.238896000
O	2.964569000	-1.414743000	6.048415000
H	3.817581000	-1.145339000	5.604141000
H	2.758595000	2.261467000	10.129144000
O	3.748444000	-1.654963000	8.525893000
O	5.007401000	1.305938000	13.885908000
O	6.678235000	0.633728000	12.383735000
O	6.462508000	1.012537000	9.550685000
O	7.767073000	0.956694000	15.357459000
H	8.768535000	0.883169000	15.340557000
O	5.579826000	-4.075977000	15.638581000
H	6.302143000	-4.764291000	15.579958000
O	10.082078000	-0.073183000	15.325731000
O	7.931869000	-5.061781000	15.428488000
O	12.552963000	-1.136933000	15.653297000
N	3.760821000	2.324667000	9.770285000
H	3.809991000	1.613814000	8.998593000
C	4.998848000	0.772641000	15.236588000
H	5.435700000	1.583012000	15.847622000
C	2.941022000	-0.792370000	15.275304000
C	1.509201000	-0.901084000	15.878191000
C	0.342544000	-0.325257000	15.055111000
H	-0.451503000	-0.044192000	15.779140000
H	-0.051704000	-1.178014000	14.457650000
C	10.692366000	-4.877678000	15.603461000
C	12.672983000	-3.497953000	15.729934000
C	12.080478000	-4.763337000	15.719876000
C	12.978456000	-0.601220000	14.389577000
H	2.936657000	1.396868000	15.427424000
H	3.523695000	-2.869260000	15.157830000
H	3.625195000	-2.183336000	16.781252000
H	5.232198000	-0.586317000	13.039429000
H	4.409350000	-0.054878000	10.897118000
H	3.330654000	0.838255000	11.992429000
H	4.572827000	2.941248000	11.590598000
H	6.469570000	3.053531000	10.020769000
H	6.739033000	2.717776000	12.458140000
H	9.039765000	1.746117000	12.537059000
H	8.870994000	1.161707000	10.854774000
H	8.840630000	2.919810000	11.197426000

C	3.565002000	0.546478000	15.743262000
H	3.585330000	0.549045000	16.850483000
C	3.824399000	-1.964052000	15.714027000
C	5.871095000	-0.462613000	15.405067000
C	5.311512000	-1.728164000	15.549166000
C	5.320298000	0.476911000	12.777606000
C	4.360480000	0.782854000	11.615864000
C	4.714702000	2.088862000	10.901522000
C	6.196820000	2.081421000	10.476560000
C	7.020992000	1.889441000	11.774495000
C	7.296571000	-0.292546000	15.389588000
C	6.168457000	-2.878103000	15.564837000
C	8.147943000	-1.417937000	15.434431000
C	7.570424000	-2.726444000	15.494586000
C	8.533200000	1.926889000	11.572337000
C	9.604855000	-1.223504000	15.411630000
C	8.422999000	-3.904562000	15.467738000
C	10.481456000	-2.426512000	15.502540000
H	3.896309000	3.252659000	9.343084000
H	6.618774000	1.357802000	8.654122000
H	10.212219000	-5.860456000	15.596512000
H	13.759209000	-3.386315000	15.827588000
H	12.702829000	-5.661801000	15.805980000
H	13.665102000	-1.301303000	13.874188000
H	13.511397000	0.339368000	14.606422000
H	12.115732000	-0.388336000	13.731661000
C	9.896167000	-3.725118000	15.512750000
C	11.895665000	-2.331066000	15.611256000
O	7.971297000	-1.238611000	10.206603000
H	7.471284000	-0.444551000	9.883331000
O	9.789756000	-0.923203000	8.288137000
H	10.581327000	-1.173398000	7.777566000
O	8.518276000	-6.934887000	10.426064000
H	7.762824000	-7.360920000	9.958798000
O	11.714953000	-4.800952000	10.221467000
H	11.286182000	-5.184515000	9.426225000
P	2.788347000	-0.796993000	7.529907000
O	3.106201000	0.673823000	7.636547000
C	1.075286000	-1.315255000	7.932040000
H	1.061176000	-2.412919000	7.804020000
H	0.412902000	-0.893411000	7.141046000

Energy Parameters in Water

Table S1. Total energies *E* of the optimized complexes of Dox and corresponding sums of Dox and SiO_2 conjugates energies

Structure	Energy Type	Energy/Hartree
	$E_{Dox} + E_{cl}$	
Dox + SiO_2 cluster		-7011.228829143
Dox + APS-SiO_2 cluster		-7109.085273875
Dox + 2APS-SiO_2 cluster		-7206.943132945
Dox + SiO_2-PMIDA cluster A		-7976.739817550
Dox + SiO_2-PMIDA cluster B		-8053.084328530
Dox + PMIDA-SiO_2 cluster (**A**) + H_2O		-8053.060162210
PMIDA-Dox (with one free P(O)-OH)		-3044.139790198
PMIDA-Dox salt A		-3044.156612084
PMIDA-Dox salt B		-3044.146658006
SiO_2-Dox complex 1	E_c	-7011.299827736
SiO_2-Dox complex 2		-7011.288183504
SiO_2-Dox complex 3		-7011.290569604
SiO_2-APS-Dox complex 1		-7109.111443578
SiO_2-APS-Dox complex 2		-7109.113112645
SiO_2-2APS-Dox complex 1		-7206.966864318
SiO_2-2APS-Dox complex 2		-7206.970503408
SiO_2-PMIDA-Dox complex 1		-7976.76317997
SiO_2-PMIDA-Dox complex 2		-7976.794151513
SiO_2-PMIDA-Dox complex 3		-7976.782308837
SiO_2-PMIDA-Dox complex 4		-8053.168681839
SiO_2-PMIDA-Dox complex 3 + H_2O		-8053.102653490
SiO_2-PMIDA-Dox salt 1		-8053.153077257
**SiO_2-PMIDA-Dox salt 2**		**-8053.169980512**
SiO_2-PMIDA-Dox salt 3		-8053.168327294

Table S2. Binding energies E_b for the optimized complexes of Dox

Structure	E_b (kJ/mol)
SiO_2-Dox complex 1	-186.41
SiO_2-Dox complex 2	-155.84
SiO_2-Dox complex 3	-162.09
SiO_2-APS-Dox complex 1	-68.71
SiO_2-APS-Dox complex 2	-73.09
SiO_2-2APS-Dox complex 1	-62.31
SiO_2-2APS-Dox complex 2	-71.86
SiO_2-PMIDA-Dox complex 1	-61.34
SiO_2-PMIDA-Dox complex 2	-142.65
SiO_2-PMIDA-Dox complex 3	-111.56
SiO_2-PMIDA-Dox complex 4	-221.46
(from SiO_2-PMIDA with one free P(O)-OH, cluster **B**)	
PMIDA-Dox (with one free P(O)-OH)	-143.88
SiO_2-PMIDA-Dox salt 1	-180.50
**SiO_2-PMIDA-Dox salt 2**	**-224.88**
SiO_2-PMIDA-Dox salt 3	-220.54
PMIDA-Dox salt A	-188.49
PMIDA-Dox salt B	-161.92

Assessment of Cytotoxicity of NCs and Microscopic Examination *In Vitro*

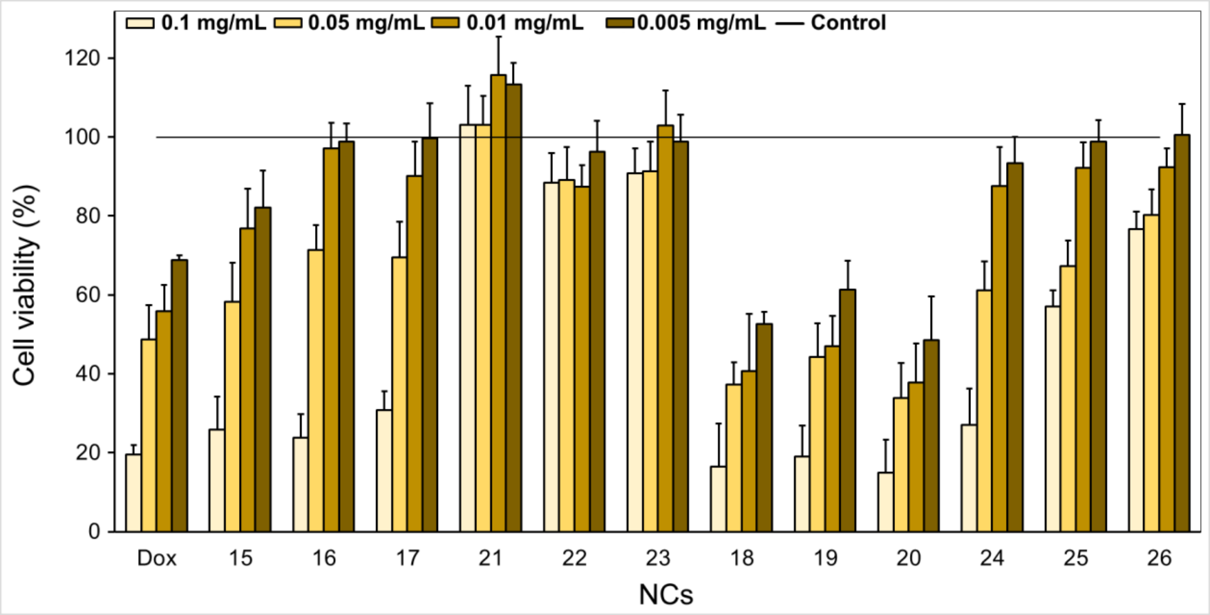

a

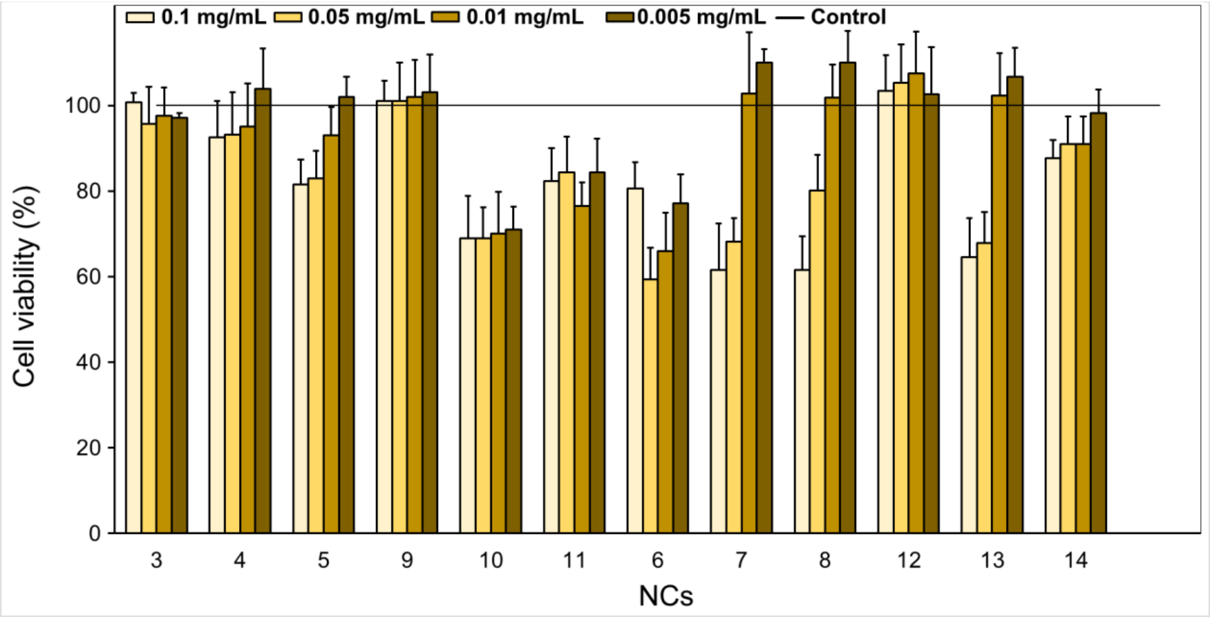

b

Figure S7. Viability of HEK-293 cells after 48-h incubation with (a) Dox-loaded NCs **15-26** and (b) NCs **3-14** (MTT assay).

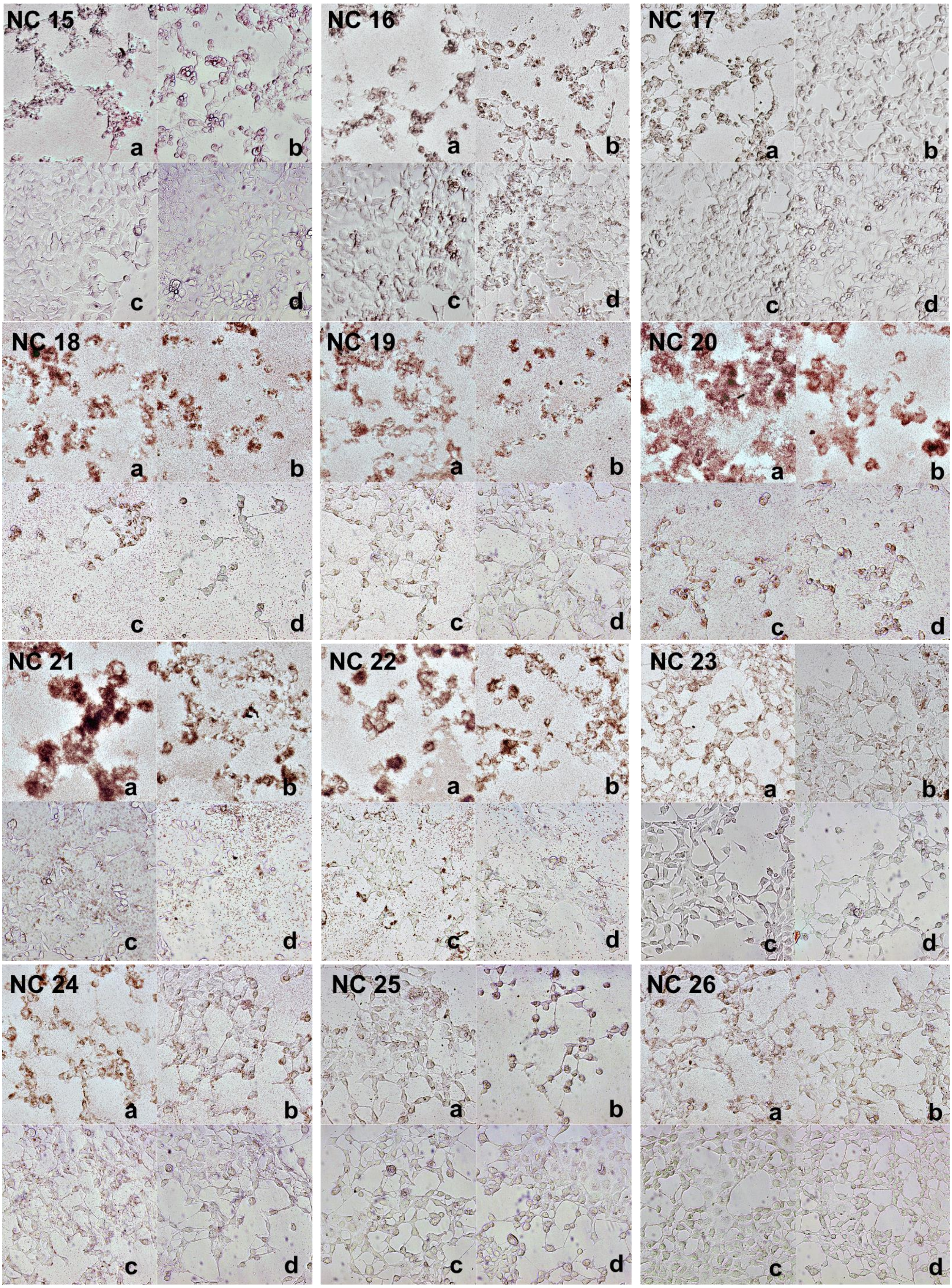

Figure S8. Optical microscope images (x200) of HEK-293 cells incubated with NCs **15–26** at concentrations of (**a**) 0.1, (**b**) 0.05, (**c**) 0.01, and (**d**) 0.005 mg/mL.

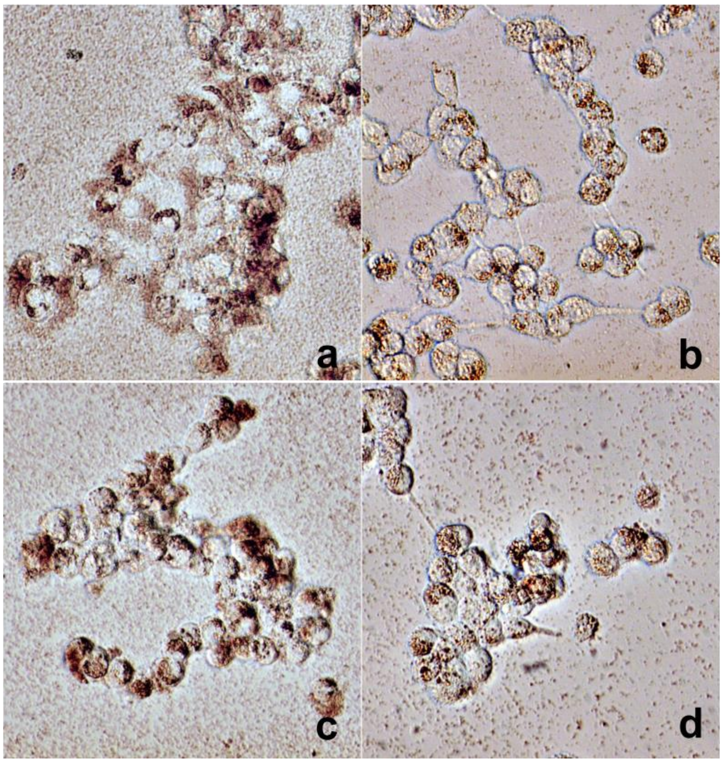

Figure S9. Optical microscope images (x400) of HEK-293 cells incubated with NC **18** at concentrations of (**a**) 0.1, (**b**) 0.05, (**c**) 0.01, and (**d**) 0.005 mg/mL.

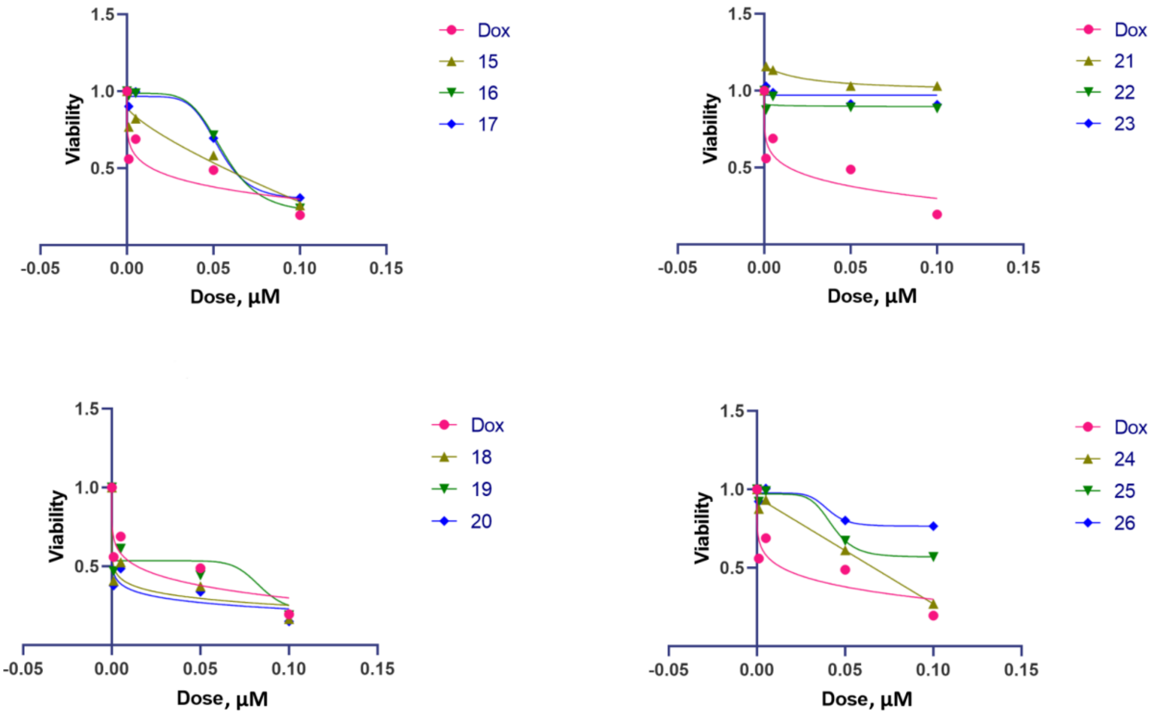

Figure S10. IC_{50} curves.
